# Fiber-orientation independent component of R_2_* obtained from single-orientation MRI measurements in simulations and a post-mortem human optic chiasm

**DOI:** 10.3389/fnins.2023.1133086

**Published:** 2023-08-25

**Authors:** Francisco J. Fritz, Laurin Mordhorst, Mohammad Ashtarayeh, Joao Periquito, Andreas Pohlmann, Markus Morawski, Carsten Jaeger, Thoralf Niendorf, Kerrin J. Pine, Martina F. Callaghan, Nikolaus Weiskopf, Siawoosh Mohammadi

**Affiliations:** ^1^Department of Systems Neurosciences, University Medical Center Hamburg-Eppendorf, Hamburg, Germany; ^2^Berlin Ultrahigh Field Facility (B.U.F.F.), Max-Delbrueck-Center for Molecular Medicine in the Helmholtz Association, Berlin, Germany; ^3^Paul Flechsig Institute – Center for Neuropathology and Brain Research, University of Leipzig, Leipzig, Germany; ^4^Department of Neurophysics, Max Planck Institute for Human Cognitive and Brain Sciences, Leipzig, Germany; ^5^Wellcome Centre for Human Neuroimaging, UCL Queen Square Institute of Neurology, University College London, London, United Kingdom; ^6^Felix Bloch Institute for Solid State Physics, Faculty of Physics and Earth Sciences, Leipzig University, Leipzig, Germany; ^7^Max Planck Research Group MR Physics, Max Planck Institute for Human Development, Berlin, Germany

**Keywords:** effective transverse relaxation rate, biophysical model, R_2_*, orientation-independent R_2_*, myelin water fraction, g-ratio, fibre dispersion, multi-echo gradient recalled echo

## Abstract

The effective transverse relaxation rate (R_2_*) is sensitive to the microstructure of the human brain like the g-ratio which characterises the relative myelination of axons. However, the fibre-orientation dependence of R_2_* degrades its reproducibility and any microstructural derivative measure. To estimate its orientation-independent part (R_2,iso_*) from single multi-echo gradient-recalled-echo (meGRE) measurements at arbitrary orientations, a second-order polynomial in time model (hereafter M2) can be used. Its linear time-dependent parameter, *β*_1_, can be biophysically related to R_2,iso_* when neglecting the myelin water (MW) signal in the hollow cylinder fibre model (HCFM). Here, we examined the performance of M2 using experimental and simulated data with variable g-ratio and fibre dispersion. We found that the fitted *β*_1_ can estimate R_2,iso_* using meGRE with long maximum-echo time (TE_max_ ≈ 54 ms), but not accurately captures its microscopic dependence on the g-ratio (error 84%). We proposed a new heuristic expression for *β*_1_ that reduced the error to 12% for *ex vivo* compartmental R_2_ values. Using the new expression, we could estimate an MW fraction of 0.14 for fibres with negligible dispersion in a fixed human optic chiasm for the *ex vivo* compartmental R_2_ values but not for the *in vivo* values. M2 and the HCFM-based simulations failed to explain the measured R_2_*-orientation-dependence around the magic angle for a typical *in vivo* meGRE protocol (with TE_max_ ≈ 18 ms). In conclusion, further validation and the development of movement-robust *in vivo* meGRE protocols with TE_max_ ≈ 54 ms are required before M2 can be used to estimate R_2,iso_* in subjects.

## 1. Introduction

The effective transverse relaxation rate (R_2_* = 1/T_2_*) is a nuclear magnetic resonance (NMR) relaxation property (Tofts, 2004) that enables non-invasive characterisation of the microstructure of the human brain (MacKay et al., 2006; Does, 2018; Weiskopf et al., 2021). The microstructural sensitivity of R_2_* makes it particularly interesting for neuroscience and clinical research studies (Langkammer et al., 2010; Draganski et al., 2011; Callaghan et al., 2014; Kirilina et al., 2020). This is because R_2_* is sensitive not only to free and myelin water pools in the brain (MacKay et al., 2006; Dula et al., 2010; Weiskopf et al., 2021) but also to microscopic perturbations in the main magnetic field ( $\vec{B_{0}}$ ) (Chavhan et al., 2009). These microscopic perturbations are caused by the different magnetic susceptibilities of biological structures (Duyn and Schenck, 2017) like the diamagnetic myelin sheath (Kucharczyk et al., 1994; Duyn, 2014; Lee et al., 2017; Alonso-Ortiz et al., 2018) and paramagnetic iron deposits in glial cells (Ordidge et al., 1994; Li et al., 2009; Yao et al., 2009). Moreover, it has been shown that R_2_* is also strongly dependent on the angular orientation of the white matter fibre tracts relative to $\vec{B_{0}}$ (Lee et al., 2011, 2012) confounding the mapping of R_2_* to the underlying microstructure. The impact of this confounding factor can be attenuated by decomposing the angular orientation dependence of R_2_* into an isotropic, i.e., angular-independent component (R_2,iso_*), and an angular-dependent component using either complex gradient-recalled echo (GRE) acquisitions at several angular orientations (Oh et al., 2013; Wharton and Bowtell, 2013; Rudko et al., 2014) or hybrid diffusion weighted imaging (DWI) and GRE acquisitions with reduced numbers of angular orientations (Gil et al., 2016). However, both methods are impractical for clinical research due to the constrained and inconvenient positioning of the patient’s head in the radiofrequency receiver coil needed to achieve the required distinct angular orientations.

A practical approach to estimate R_2,iso_* was recently proposed by Papazoglou et al. (2019). They showed that R_2,iso_* can be estimated from the magnitude signal of a single multi-echo GRE (meGRE) measurement using a second-order model in time hereafter denoted as M2. The model was derived from a two-pool system based on the hollow cylinder fibre model (HCFM) (Wharton and Bowtell, 2012, 2013). In M2, the linear component in time (*β*_1_) is a proxy for R_2,iso_* and the orientation-dependent part is regressed out by the second-order term in time (*β*_2_). Although M2 is just an approximation of the original HFCM multi-compartment model and thus less accurate, it is, to our knowledge, the only way of estimating R_2,iso_* from magnitude-only meGRE data with a single orientation of the head.

Another advantage of the M2 is its relation to the HCFM model, allowing for direct translation of the M2-proxy for R_2,iso_* (i.e., the *β*_1_ parameter) into microscopic tissue properties. However, a drawback of this model is the assumption in M2 that the signal contribution of the myelin water can be neglected, limiting the microscopic interpretability of the estimated *β*_1_ parameter. For example, the M2-based prediction of *β*_1_ depends only on the transverse relaxation rate of the free water molecules of the non-myelinated compartments (R_2N_) and thus is independent of any changes associated with the myelin water signal or the myelin water fraction (MWF). This model’s prediction could contradict experimental observations reporting that R_2_* (and presumably R_2,iso_*) is linearly dependent on MWF (see Lee et al., 2017; Kirilina et al., 2020; Milotta et al., 2023). Moreover, M2 assumes that axonal fibres are perfectly aligned or even described by one representative axon. However, most of the fibre bundles in the human brain possess a diverse range of topographies, i.e., show fanning and bending, or mildly to acute crossing (e.g., Schmahmann et al., 2007, 2009; Jeurissen et al., 2019) and different levels of relative myelination (e.g., Mohammadi et al., 2015). Besides that, the performance of M2 in estimating R_2,iso_* via *β*_1_ has only been tested with data acquired at very long maximum echo times up to ≈ 54 ms (Papazoglou et al., 2019). Such a long maximum echo time is unusual for *in vivo* meGRE measurements with whole-brain coverage (Weiskopf et al., 2013; Ziegler et al., 2019), because it increases the total scan time as well as the propensity for bulk and physiological motion.

This work explores the potential and pitfalls of using M2 to estimate R_2,iso_* via *β*_1_, from a single-orientation meGRE, while varying biological fibre properties and maximum echo times. To this end, we use simulated (hereafter *in silico*) data and *ex vivo* MRI. The *in silico* data are simulated using a three-pool system based on the HCFM to generate realistic meGRE datasets from an ensemble of myelinated axons, for which the ground truth biophysical parameters (i.e., g-ratio, fibre dispersion and angular orientation) are known and can be varied. The *ex vivo* dataset combines high-resolution DWI and multi-orientation meGRE imaging of a human optic chiasm to generate gold-standard datasets where the fibre orientation and dispersion can be estimated. Both datasets are used to perform the following analyses: First, we assess the performance of M2 to estimate R_2,iso_* via *β*_1_ for varying g-ratio values and fibre dispersions. Second, we assess the microstructural interpretability of *β*_1_. To this end, we test the model-prediction of M2 that *β*_1_ is independent of MWF by evaluating the deviation between the biophysically predicted *β*_1_ by M2 and the fitted *β*_1_ using the *in silico* data. Additionally, we perform the same comparison to the fitted *β*_1_ as above using a novel heuristic expression that incorporates the MWF dependence into the predicted *β*_1_. Third, we use the heuristic expression for *β*_1_ to calculate MWF from the *β*_1_ of the *ex vivo* data for two sets of compartmental R_2_ values, i.e., *in vivo* and *ex vivo* configurations. And fourth, we assess the performance of M2 to estimate R_2,iso_* via *β*_1_ for different echo times ranges.

## 2. Background

### 2.1. Overview of the hollow cylinder fibre model and the approximated log-quadratic model

The HCFM (Wharton and Bowtell, 2012, 2013) proposes an analytical approximation describing the dependence of the GRE signal on the angular orientation ( $\theta_{\vec{\mu }}$ ) defined as the angle between the main magnetic field $\vec{B_{0}}$ and the hollow-cylinder fibre ( $\vec{\mu }$ ). This approximation establishes that the total MR signal comes from water molecules in an *infinitely long* and perfectly aligned hollow cylinder affected by the diamagnetic myelin sheath (Liu, 2010). The diamagnetic myelin sheath magnetically perturbs the water molecules in three distinct compartments: (1) the intra-axonal (S_A_), (2) myelin (S_M_) and (3) extra-cellular (S_E_) compartments (details in Supplementary material, section 2). When the signal of the water molecules in the myelin compartment is neglected (i.e., at long echo times: TE > T_2_ of myelin, T_2M_), the signal magnitude of the HCFM can be approximated by a log-quadratic model (M2) in time (Papazoglou et al., 2019):


(1) $$\mathrm{M2}:\ln\left(\left|S_{N}\left(t,\theta_{\vec{\mu }}\right)\right|\right)\approx \beta_{0}-\beta_{1}t-\beta_{2}\left(\theta_{\vec{\mu }}\right)t^{2},$$

where $\beta_{0,1,2}$ are the model parameters. In this model, the slope *β*_1_ is considered as a proxy for R_2,iso_* because it does not possess any $\theta_{\vec{\mu }}$ dependence, whereas *β*_2_ contains all the $\theta_{\vec{\mu }}$ dependent information of R_2_* (detailed derivation can be found in section 4, Supplementary Equations S17b,c).

Classically, R_2_* is estimated by the slope (*α*_1_) of the log-linear model (M1) (Elster, 1993):


(2) $$M1:\ln\left(\left|S\left(t\right)\right|\right)\approx \alpha_{0}-\alpha_{1}\left(\theta_{\vec{\mu }}\right)t$$

where *α*_1_ is a function of R_2,iso_* and the $\theta_{\vec{\mu }}$ dependent components of R_2_* (e.g., see Lee et al., 2011, 2012).

In this model, the offset parameter $\alpha_{0}$ captures a large portion of the remaining information like contrast parameters, e.g., magnetisation transfer and longitudinal relaxation rate; and experimental parameters, e.g., transmit field. For M2, we assume that $\beta_{0}$ behaves in an identical fashion, i.e., this parameter captures all the remaining information as $\alpha_{0}$ , even though this assumption is not explicitly shown in the HCFM [see Discussion in Wharton and Bowtell (2013)].

### 2.2. Myelin independence of *β*_1_ parameter as predicted by the log-quadratic model (M2)

The slope *β*_1_ of M2, which is a proxy for R_2,iso_*, is derived from the HCFM by assuming a two-pool system in the slow-exchange regime: a fast decaying water pool consisting of the myelin water with a relaxation rate $R_{2M}$ and a non-myelin water pool with a relaxation rate $R_{2N}=R_{2A}=R_{2E}$ . In this work, we assumed that this non-myelin water pool consisted of the intra and extra cellular water, based on the findings and simplifications of Dula et al. (2010) and Wharton and Bowtell (2013). The only source of dephasing in the HCFM is caused by the magnetic properties of the hollow-cylinder fibre. All potential other perturbers are ignored (e.g., non-local effects of susceptibility inhomogeneities due to cavities, vessels, iron molecules, and diffusion) as well as other anisotropic magnetic properties, e.g., the magnetisation transfer effects (Pampel et al., 2015), influencing transverse relaxation rate (Knight et al., 2017; Birkl et al., 2021; Tax et al., 2021) or longitudinal relaxation rate (Labadie et al., 2014; Schyboll et al., 2019; Chan and Marques, 2020; Kleban et al., 2021). In white matter, this simplification seems to be reasonable since the HCFM describes the orientation dependence of the meGRE signal to a great extend (Wharton and Bowtell, 2012). Consequently, in the approximation of M2 (Eq. 1), the predicted $\beta_{1}$ parameter (hereafter $\beta_{1,nm}$ , where nm represents ‘no myelin contribution’) is given by the transverse relaxation rate of the non-myelin water pool ( $R_{2N}$ ):

(3) $$\beta_{1}\approx \beta_{1,nm}=R_{2N}.$$

We hypothesise here that for realistic tissue composition where the myelin compartment cannot be neglected (i.e., g-ratio equal to or smaller than 0.8), Eq. 3 is invalid. This hypothesis is supported by previous observations showing that R_2_* (and presumably R_2,iso_*) depends on the myelin water fraction, MWF (e.g., Lee et al., 2017; Weber et al., 2020; Milotta et al., 2023).

Here, we propose an alternative heuristic biophysical expression of the predicted *β*_1_ parameter (hereafter $\beta_{1,m}$ , where m denotes ‘with myelin contribution’):

(4) $$\beta_{1,m}=\frac{\rho_{N}R_{2N}\left(1-V_{M}\right)+V_{M}\rho_{M}R_{2M}}{\left(\rho_{N}\left(1-V_{M}\right)+V_{M}\rho_{M}\right)},$$

under the assumption that the proton densities of the non-myelinated compartments are equal (i.e., ρ_A_ = ρ_E_ ≡ ρ_N_) and the volume of the non-myelinated compartment is defined as one minus the myelin compartment’s volume (= 1 – V_M_). The heuristic expression in Eq. 4 can be analytically derived under the condition that TE < T_2_ of the myelin compartment based on the HCFM (details can be found in section 4, Supplementary Equation S18).

In this case, $\beta_{1,m}$ can be re-written as a function of the myelin water fraction (MWF, Supplementary Equation S19a, section 4), R_2N_ and R_2M_:

(5) $$\beta_{1}\approx \beta_{1,m}=\left(1-\mathrm{MWF}\right)R_{2N}+\mathrm{MWF}\cdot R_{2M}.$$

Consequently, the MWF can be calculated by re-ordering Eq. 5 as a function of the R_2_’s values:

(6) $$MWF=\frac{\beta_{1,m}-R_{2N}}{R_{2M}-R_{2N}}.$$

Based on our hypothesis, we expect that the heuristic expression for $\beta_{1,m}$ can better describe the fitted *β*_1_ when varying the g-ratio, and thus is a better proxy of R_2,iso_*.

## 3. Materials and methods

This section explains the approaches used for data acquisition, data analysis and for comparing the results obtained from the *ex vivo* data and the findings derived from the *in silico* data.

### 3.1. *Ex-vivo*: optic chiasm

#### 3.1.1. Sample and data acquisition

A human optic chiasm (OC) from a patient without any diagnosed neurological disorder was measured (male, 59 years, multi-organ failure, 48 h *postmortem* interval, ~80 days of fixation in phosphate buffered saline (PBS) pH 7.4 with 0.1% sodium acide NaN_3_ containing 3% paraformaldehyde +1% glutaraldehyde) with prior informed consent (Ethical approval #205/17-ek). Two MR techniques were used: multi-echo GRE (meGRE) and diffusion-weighted MRI (dMRI). The meGRE data used here have been in parts used already in Papazoglou et al. (2019).

**Figure 1:**
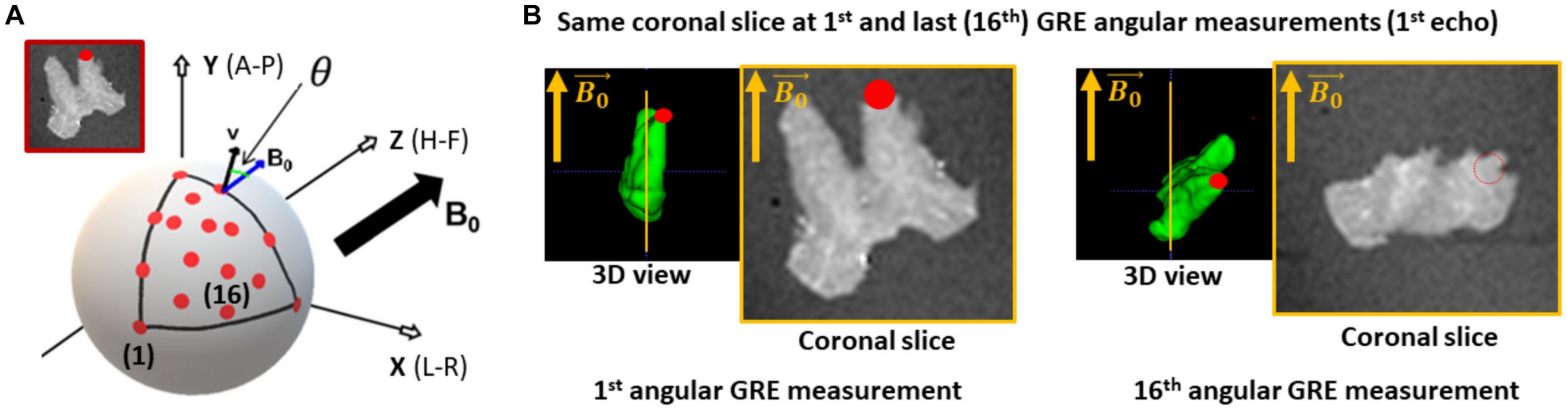
Acquisition of the multi-angular multi-echo gradient recalled echo (meGRE) *ex vivo* data. **(A)** An illustration of the different angular measurements performed on the optic chiasm (OC) specimen. The red dots show the position of the optical tracts (see inset) for the different measurements. The different coordinates (spatial, x-y-z and anatomical, anterior-head-right, A-H-R) are shown (adapted illustration from Papazoglou et al., 2019). **(B)** Illustration of the first echo meGRE image acquired at the first and last angular measurement. The 3D view shows the specimen position to the main magnetic field $\vec{B_{0}}$ and the position of the optical tract (red dot). The yellow line shows the same coronal slice image.

All meGRE acquisitions were performed on a 7 T Siemens Magnetom MRI scanner (Siemens Healthcare GmbH, Erlangen, Germany) using a custom 2-channel transmit/receive circularly polarised (CP) coil with a diameter of 60 mm. The OC sample was fixed within an acrylic sphere of 60 mm diameter filled with agarose (1.5% Biozym Plaque low melting Agarose, Merck, Germany) dissolved in PBS (pH 7.4 + 0.1% sodium acide) and scanned at sixteen orientations (covering a solid angle, with azimuthal and elevation angles from 0° to 90°, Figure 1A) using the 3D meGRE MRI (hereafter: **GRE dataset**). For each angular meGRE measurement (Figure 1B), sixteen echoes were acquired at equally spaced echo times (TE) ranging from 3.4 to 53.5 ms (increment 3.34 ms) with a repetition time (TR) of 100 ms, a field of view (FoV) of (39.00 mm)^3^, a matrix size of 112^3^, resulting in an isotropic voxel resolution of (0.35 mm)^3^, non-selective RF excitation with a flip angle of 23° and a gradient readout bandwidth of 343 Hz/px.

The multi-shell dMRI data (hereafter: **dMRI dataset**), suitable for NODDI analysis, were acquired, three months later, on a 9.4 T small animal MR system (Bruker Biospec 94/20; Bruker Biospin, Ettlingen, Germany) using a 2-channel receiver cryogenically cooled quadrature transceiver surface RF coil (Bruker Biospin, Ettlingen, Germany) and a gradient system with G_max_ = 700 mT/m per gradient axis. This dataset was acquired with a slice-selective (2D) pulsed-gradient spin-echo (PGSE) technique, consisting of four diffusion-weighting shells (number of directions) of b = 1,000 s/mm^2^ (60), 4,000 s/mm^2^ (60), 8,000 s/mm^2^ (60) and 12,000 s/mm^2^ (60) with 35 non-diffusion-weighted volumes (~ 0 s/mm^2^). The fixed diffusion parameters were diffusion time Δ = 13 ms, diffusion gradient duration δ = 6 ms. The remaining sequence parameters were TE = 27 ms, TR = 30 s (to acquire all the slices), FoV = 20.75 × 16.00 × 12.50 mm^3^, matrix size = 83 × 64 × 50, isotropic voxel resolution = (0.25 mm)^3^, slice selective pulses with flip angles of 90° (excitation) and 180° (refocusing) and receiver bandwidth of 9,411 Hz/px.

Note that we used different MR systems for dMRI and meGRE measurements, because it was intended to use the optimal system for the respective measurement. The dMRI dataset was acquired on a Bruker Biospec with cryo-coil to take advantage of the scanner’s high gradient strength, allowing for acquisition of high-resolution images at optimal b-values for *ex vivo* tissue [up to 12,000 s/mm^2^ (Roebroeck et al., 2018)]. Moreover, we used a TR of 30 s for dMRI measurements to ensure full magnetisation recovery and to reduce possible biases for diffusion analysis (section 3.1.2). The meGRE data was acquired on a human 7 T Siemens Magnetom MRI scanner because an optimised meGRE sequence was available on this system, including a self-built *ex vivo* sample coil.

Since the tissue sample was already fixed and immersed in PBS and sodium acide solution to preserve the tissue quality (Minassian and Huang, 1979), the time between the acquisitions (less than 2 weeks) did not affect the tissue quality.

#### 3.1.2. Dispersion and mean fibre orientation estimation from dMRI dataset

To incorporate the voxel-wise information regarding the angular orientation of the fibres to $\vec{B_{0}}$ and fibre’s dispersion, the dMRI datasets were corrected using a simple rigid-body registration to remove a potential drift of the sample during measurements. The dMRI data were analysed with two diffusion models: Neurite Orientation Dispersion and Density Imaging (NODDI) (Zhang et al., 2012) and Diffusion Tensor Imaging (DTI) (Basser et al., 1994). The NODDI toolbox was adjusted for *ex vivo* analysis (Wang et al., 2019) and used all the diffusion shells. The main neurite (hereafter fibre) orientation ( $\vec{\mu }$ ), a measure of the fibre dispersion (κ), and fibre density (volume fraction of the intracellular compartment, ICVF) maps were estimated from this analysis. The DTI model used the first two diffusion shells (b-values: 1000 s/mm^2^ and 4,000 s/mm^2^) and was used only for estimating the fractional anisotropy (FA) map, which in turn was used only for diffusion-to-GRE coregistration (section 3.1.3). Note that eddy currents were small in this dMRI data because the data were acquired with a small FoV in the gradient-coil centre using the standard Bruker gradient coil. Moreover, the cryo-coil provided sufficiently high SNR values for unbiased dMRI model parameters (the mean SNR across the specimen was approximately 57 for the b-value = 0 s/mm^2^ images). The SNR was calculated by dividing the MR signal by the standard deviation of the background voxels of its corresponding image (Kellman and McVeigh, 2005).

#### 3.1.3. Coregistration of the GRE angular measurements and dMRI results

**Figure 2:**
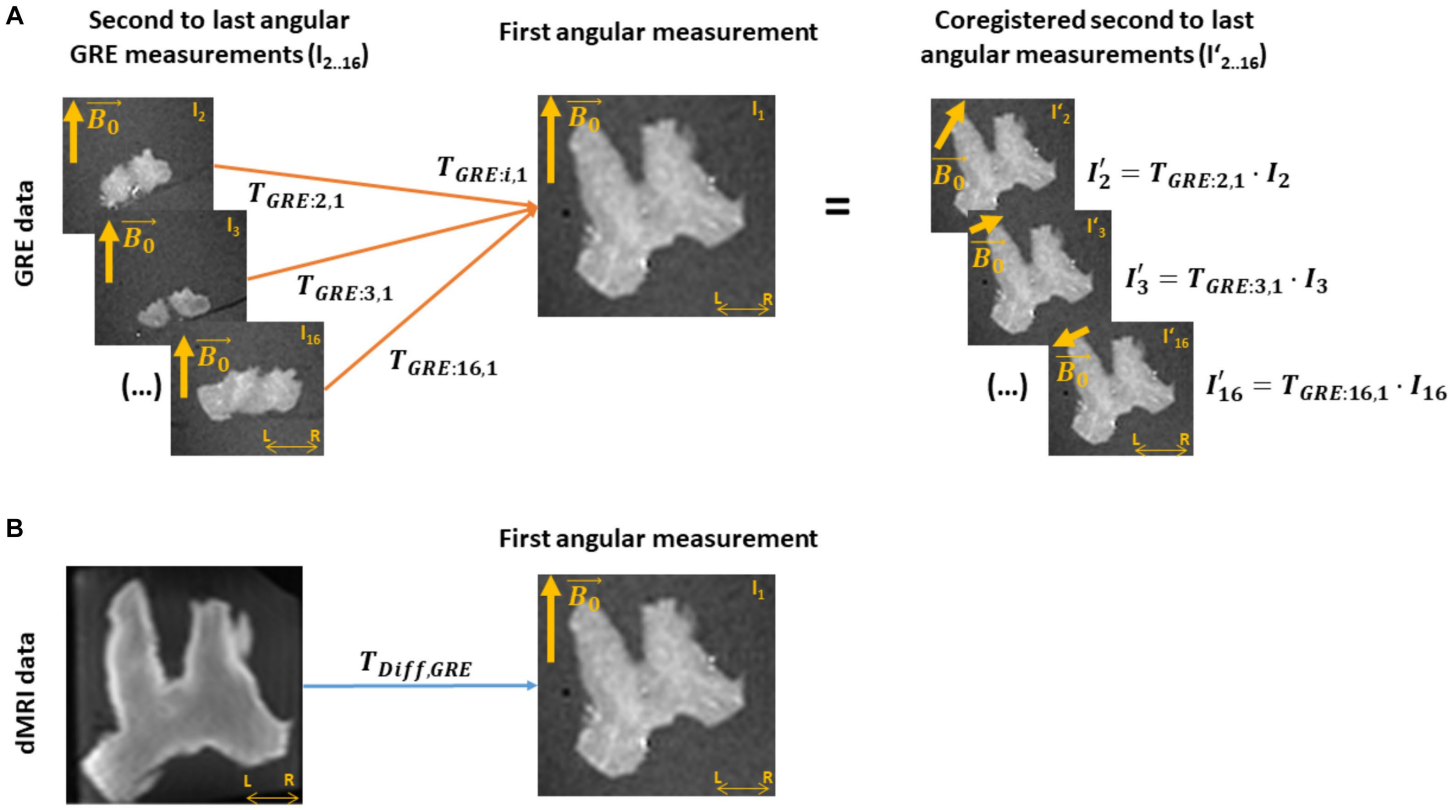
Coregistration of the *ex vivo* GRE and dMRI measurements. **(A)** A transformation matrix (T_GRE_) is obtained by coregistering all other multi-echo gradient-recall-echo (meGRE) datasets (I_2.0.16_) to the first measurement (I_1_, T_GRE: i,1_). This transformation matrices not only align, voxel-wise, the images of the meGRE datasets (I’_2.0.16_) to the first dataset, but also adjusts the directions of the main magnetic field ( $\vec{B_{0}}$ ) per angular measurement to preserves their relative orientation with respect to the first meGRE dataset. **(B)** A transformation matrix (T_Diff,GRE_) is obtained by coregistering the diffusion MRI (dMRI) image to the first angular GRE measurement. This transformation will allow the coregistration of the NODDI analysis results to the GRE data.

To establish a voxel-to-voxel relationship between the meGRE signal at different angular orientations and the properties estimated from dMRI, i.e., κ, $\vec{\mu }$ and ICVF, we coregistered the angular meGRE measurements and the dMRI measurement. To this end, we estimated two sets of transformation matrices: first, transformation matrices that coregister the i-th angular measurements in GRE space, $T_{GRE:i,1}$ (with i = 2… 16, see Figure 2A); and second, a transformation matrix that coregisters from GRE space to dMRI space, $T_{Diff,GRE}$ (see Figure 2B). The coordinate system of GRE space was defined by the first meGRE angular measurement. This reference was chosen due to the alignment of the optical nerves to $\vec{B_{0}}$ and following the procedure adopted in a previous study (Papazoglou et al., 2019). The meGRE coregistration and estimation of $T_{GRE:i,1}$ were performed using the 3D Slicer software^1^ (Fedorov et al., 2012), while the GRE-to-diffusion transformation was performed using the coregistration module in SPM 12.^2^

#### 3.1.4. Voxel-wise estimation of the angular orientation, $\theta_{\vec{\mu }}$ , between fibres and $\vec{B_{0}}$

**Figure 3:**
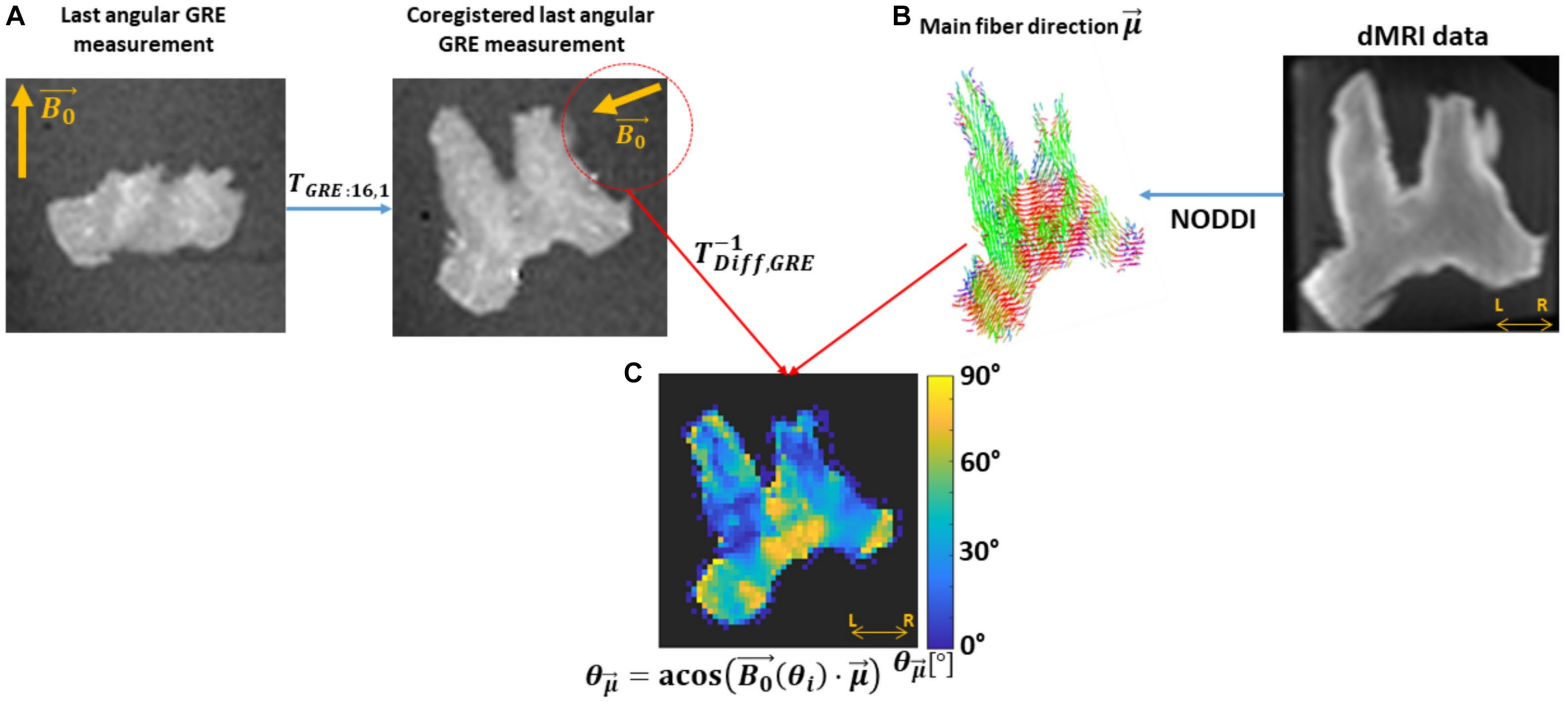
Estimation of the voxel-wise angular $\theta_{\vec{\mu }}$ map. This estimation needed the B_0_ direction per angular GRE measurement ( $\vec{B_{0}}\left(\theta_{i}\right)$ ) in diffusion space and the main fibre direction. **(A)** The $\vec{B_{0}}\left(\theta_{i}\right)$ was estimated by applying to $\vec{B_{0}}$ , first, the transformation matrix between GRE volumes (T_GRE:i,1_) and later from GRE-to-diffusion (T^−1^_Diff,GRE_). **(B)** The main fibre direction ( $\vec{\mu }$ ) was acquired by analysing the dMRI data with the NODDI model. **(C)** Then, the voxel-wise $\theta_{\vec{\mu }}$ per angular measurement was computed by the arccosine of the scalar product between the projected $\vec{B_{0}}\left(\theta_{i}\right)$ and the main diffusion direction ( $\vec{\mu }$ ), $\theta_{\vec{\mu }}=acos\left(\vec{B_{0}}\left(\theta_{i}\right)•\vec{\mu }\right)$ . This sketch shows the steps for the last GRE angular measurement.

The angular orientation $\theta_{\vec{\mu }}$ between fibres and $\vec{B_{0}}$ for each meGRE angular measurement was calculated in dMRI space and the resulting $\theta_{\vec{\mu }}$ maps mapped onto GRE space. For that, the arccosine of the inner product between $\vec{B_{0}}\left(\theta_{i}\right)$ and $\vec{\mu }$ , i.e., $\theta_{\vec{\mu }}=arccos\left(\vec{B_{0}}\left(\theta_{i}\right)•\vec{\mu }\right)$ was computed (Figure 3C). In this computation, $\vec{B_{0}}\left(\theta_{i}\right)$ is the resulting $\vec{B_{0}}$ after the transformation from the i-th meGRE angular measurement to the first meGRE angular measurement ( $T_{GRE:i,1}$ ), and the transformation from GRE to dMRI space ( $T_{Diff,GRE}^{-1}$ ) (Figure 3A). The main fibre direction was obtained by the $\vec{\mu }$ map from the NODDI analysis (Figure 3B).

Note that $\theta_{\vec{\mu }}$ was computed in dMRI space instead of GRE space to avoid undersampling and interpolation caused by transforming the dMRI-based $\vec{\mu }$ to GRE space. These sources of error do not occur by transforming $\vec{B_{0}}$ to dMRI space, i.e., computing $T_{Diff,GRE}^{-1}\cdot T_{GRE:i,1}\cdot \vec{B_{0}}$ , for each GRE angular measurement, since it is a global rather than a per-voxel measure. Finally, the $\theta_{\vec{\mu }}$ maps together with the ICVF and κ maps (not shown in Figure 3) were transformed using $T_{Diff,GRE}$ . Exemplary $\theta_{\vec{\mu }}$ maps in GRE space are shown in Supplementary Figure S1 (first row).

#### 3.1.5. Masking and pooling the *ex vivo* data

Before analysis, the *ex vivo* data required further pre-processing to remove outliers and to ensure a robust assessment of the effect of fibre dispersion and $\theta_{\vec{\mu }}$ on R_2_*. For that, the *ex vivo* data were masked using the coregistered ICVF map and later pooled across the sixteen coregistered meGRE angular measurements.

In this process, all voxels in the OC with an ICVF >0.8 were selected and pooled across all the meGRE angular measurements, hereafter referred to as cumulated data. The ICVF threshold was used because the extra-axonal space in the *ex vivo* specimen is reduced (e.g., Stikov et al., 2011). The application of this threshold reduced the number of voxels in the OC by 7.2% (~ 600 over 8,744 voxels). By pooling the data, the resulting cumulated data dependent on TE but also on $\theta_{\vec{\mu }}$ from 0° to 90°, and on fibre dispersion assessed by κ from 0 to 6.

### 3.2. Simulated R_2_* signal decay from the HCFM

Multi-echo GRE signal decay was simulated as ground truth (hereafter, *in silico* data) to assess the impact on M2 of variable fibre orientation, dispersion and myelination (i.e., g-ratio). For that, we estimated averaged MR signals calculated from an ensemble of 1,500 hollow cylinders. The cylinders were evenly distributed on a sphere with defined spherical coordinates: an azimuthal angle φ rotating counter-clockwise from 0° to 359° starting aligned with the +X axis, and elevation angle θ rotating from 0° (+Z) to 180° (−Z). The signal contribution per hollow cylinder was modelled using the frequency-averaged signal equations from the HCFM for all the compartments including the myelin compartment (Supplementary Equations S1, S3, section 2).

**Figure 4:**
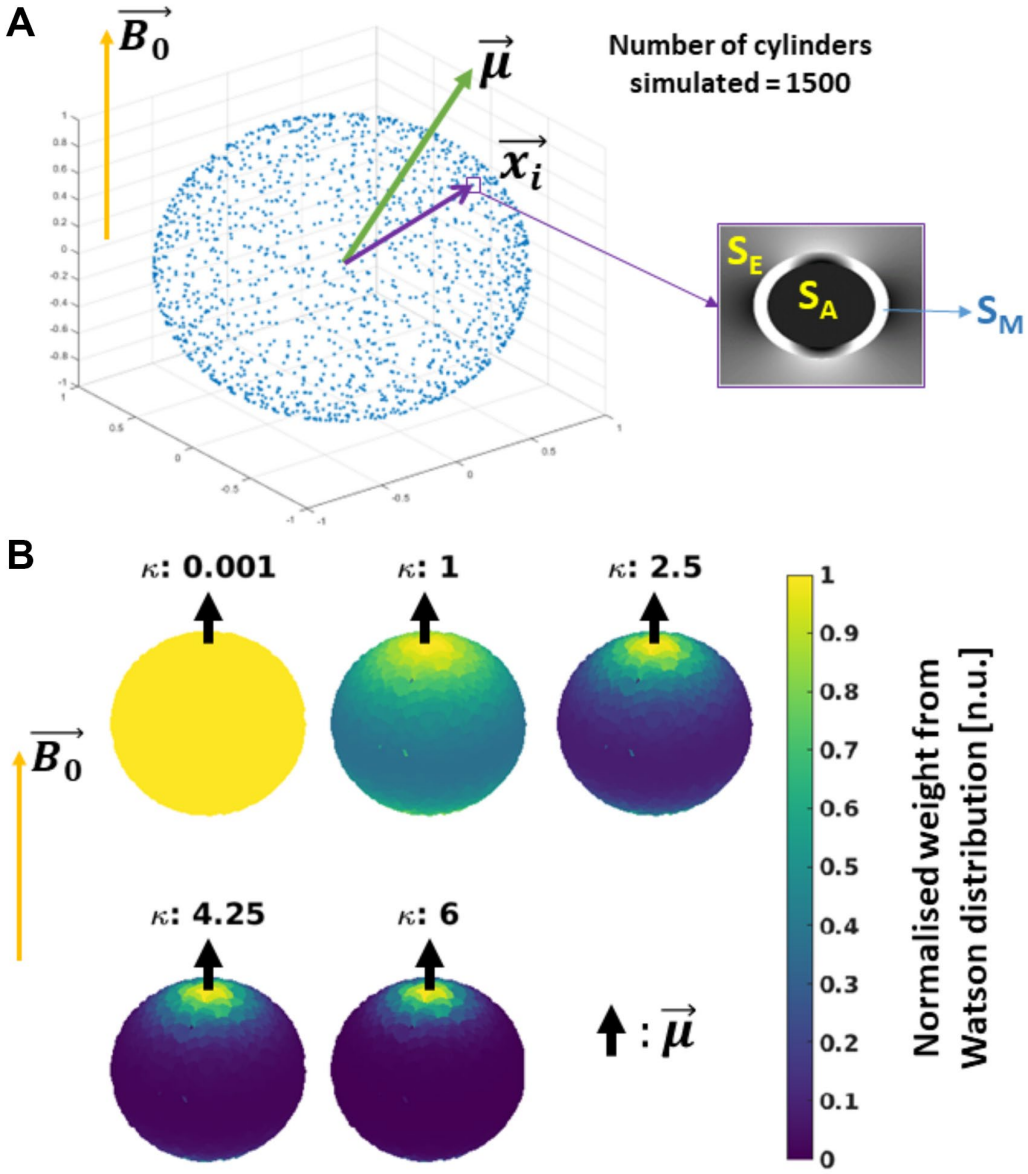
Schematics of the simulated *in silico* data: **(A)** Simulation: 1500 hollow cylinders, each of them defined by the vector $\vec{x_{i}}$ , were distributed evenly on a sphere (see the blue dots). A mean orientation $\vec{\mu }$ of the cylinders is defined, with the external magnetic field ( $\vec{B_{0}}$ ) oriented parallel to the Z-axis. The signal contribution per cylinder was modelled using the Hollow Cylinder Fibre Model (HCFM) with the intra-axonal (S_A_), extra-axonal (S_E_) and myelin (S_M_) compartments (inset). **(B)** Addition of cylinder’s dispersion: the dispersion effect was added by weighting the signal coming from the cylinders by the parameter κ from the Watson distribution and $\vec{\mu }$ (Eq. 8b). The parameter κ is limited from κ = 0 for isotropically dispersed to κ = infinity to fully parallel fibres. Here, $\vec{\mu }$ is parallel to $\vec{B_{0}}$ .

In the simulation framework, three assumptions were made. First, the $\vec{B_{0}}$ was fixed and oriented parallel to +Z (Figure 4A). Second, the approximated piece-wise function of D_E_ in the S_E_ signal was replaced by its analytical solution (Supplementary Equations S2b, S4, S7, S8, section 3; respectively), because a discontinuity in this piece-wise function was observed at the so-called critical time (Yablonskiy and Haacke, 1994; Wharton and Bowtell, 2013). See section 3 in Supplementary material for a detailed discussion. And third, we ignored the near-field effects between cylinders, therefore the total signal is the sum of all the complex signals from each cylinder as defined in Supplementary Equations S1–S3.

To incorporate the effect of fibre dispersion in the *in silico* data, the ensemble-average signal was calculated by weighting S_c_ with the Watson distribution (*W*) (Sra and Karp, 2013 and Eq. 8b). This weight from the Watson distribution was calculated using the position of each simulated cylinder, $\vec{x_{i}}$ , and a mean fibre orientation $\vec{\mu }$ , both defined with spherical coordinates (φ, θ) and ( $\phi_{\vec{\mu }}$ , $\theta_{\vec{\mu }}$ ), respectively (Figure 4A). For simplification, $\vec{\mu }$ was restricted to an azimuthal angle of zero ( $\phi_{\vec{\mu }}$ = 0°). Then, the analytical expression of the ensemble-average signal, S_W_, is defined as follows:


(7a) $$S_{W}\left(\kappa ,t,\theta_{\vec{\mu }}\right)=\frac{\sum_{i}\left(S_{C}\left(t,\theta_{i}\right)\left[W\left(\kappa ,\theta_{\vec{\mu }},\phi_{i},\theta_{i}\right)\right]\right)}{\sum_{i}W\left(\kappa ,\theta_{\vec{\mu }},\phi_{i},\theta_{i}\right)},$$

(7b) $$\;\mathrm{where}\;W\left(\kappa ,\theta_{\vec{\mu }},\theta_{i},\phi_{i}\right)=C_{1}{\left(\frac{1}{2},\frac{3}{2},\kappa \right)}^{-1}e^{\kappa {\left(\vec{\mu }\left(\theta_{\vec{\mu }}\right)\cdot \vec{x_{i}}\left(\phi_{i},\theta_{i}\right)\right)}^{2}}.$$

In Eq. 7b, $C_{1}$ () is the confluent hypergeometric function, which is the normalisation factor of the Watson distribution, and the exponent holds the norm of the inner product between each individual i-th cylinder $\vec{x_{i}}$ and $\vec{\mu }$ . The level of dispersion was modulated by the parameter κ (Zhang et al., 2012; Sra and Karp, 2013) as shown in Figure 4B for a few cases. It is important to note that the notation $\theta_{\vec{\mu }}$ for the elevation angle of $\vec{\mu }$ used here is equal to the one used to describe the fibre’s angular orientation in the *ex vivo* data (section 3.1.4). This is intentional since they stand for the same concept in both datasets. This simulation approach was used in previous conference publications [Fritz et al. (2020, 2021)].

With the ensemble averaged signal equation (Eq. 7a), a meGRE signal decay can be computed based on the relevant parameters listed in the following Table 1.

We tested the validity of M2 and its microstructural derivatives like MWF (Eq 6) for varying microstructural parameter settings. This included two different sets of compartmental R_2_ values (i.e., R_2N_ and R_2M_) (Dula et al., 2010; Wharton and Bowtell, 2013). The sets represent two possible extremes of compartmental R_2_ values at 7 T: (1) *ex vivo* compartmental R_2_ values measured from an *ex vivo* rat spinal cord with similar tissue preparation procedure as the optic chiasm in this work [i.e., fixed with 4% PFA and hydrated in PBS (Dula et al., 2010)], and (2) *in vivo* compartmental R_2_ values obtained from *in vivo* human measurements (Wharton and Bowtell, 2013). The *ex vivo* compartmental R_2_ values are reported in the main manuscript whereas the *in vivo* compartmental R_2_ values are reported in the Supplementary material, Section 7.

Finally, each simulated meGRE signal decay was replicated 5,000 times with an additive Gaussian complex noise (Gudbjartsson and Patz, 1995) to approximate the SNR of the experimental *ex vivo* data (see Supplementary material, section 5). The experimental SNR was calculated by dividing the MR signal acquired at the first echo by the standard deviation of the background voxels of its corresponding image (Kellman and McVeigh, 2005), resulting in a mean SNR across the selected voxels of the OC of 112.

This simulation framework is publicly and freely available in Github.^3^

### 3.3. Data analysis

#### 3.3.1. Data fitting and binning

The *ex vivo* data (section 3.1) and *in silico* data (each of 5,000 replicas per simulated meGRE signal decay, section 3.2) were analysed with the log-linear and log-quadratic models, M1 (Eq. 2) and M2 (Eq. 1), respectively. In both models, the *α*’s (*α*_0_ in arbitrary units, *α*_1_ in units of 1/s) from M1, and *β*’s (*β*_0_ in arbitrary units, *β*_1_ in units of 1/s and *β*_2_ in units of 1/s^2^) from M2, hereafter referred to as the *α*-parameters and *β*-parameters, were estimated. To fit the data, ordinary Least Square (OLS) optimization was used for both models in custom-made Matlab code. Three fittings were performed using three different meGRE subsets, that varied by their maximum TE (TE_max_) values: TE_max_ = 54 ms (all 16 time points), TE_max_ = 36 ms (first 10 points) and TE_max_ = 18 ms (first 5 time points). The first meGRE subset with TE_max_ of 54 ms replicated the meGRE protocols of the *ex vivo* studies, while the meGRE subset with TE_max_ of 18 ms could be considered as a typical meGRE protocol for *in vivo* studies [at least with regards to the sample size and TE range used in the multi-parametric mapping protocol (Weiskopf et al., 2013)]. The meGRE subset with TE_max_ of 36 ms was chosen as an intermediate subset between both protocols.

**Figure 5:**
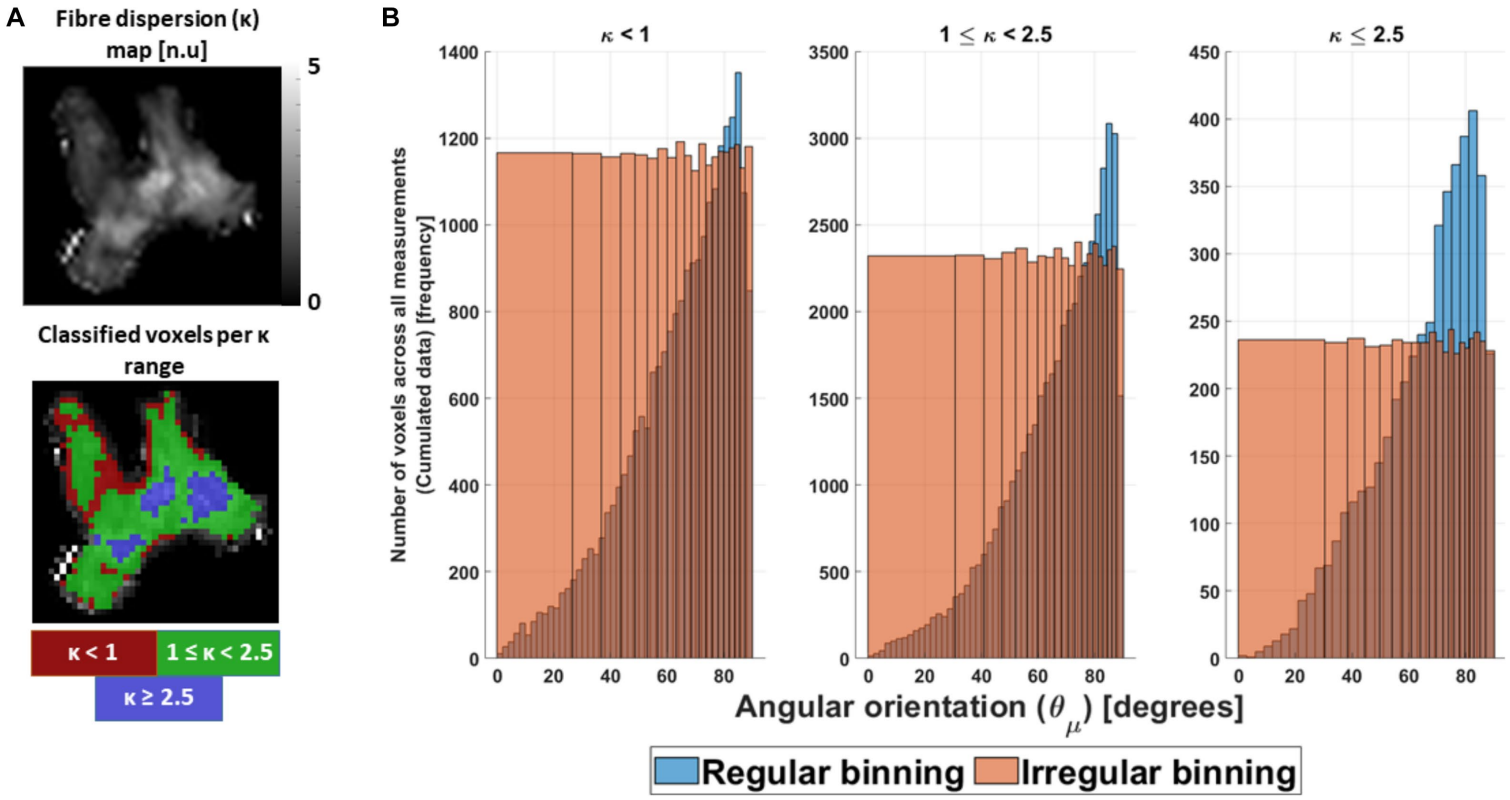
Preparation of the *ex vivo* data for analysis. **(A)** The cumulated *ex vivo* data were distributed first as a function of κ parameter, to ensure similar fibre dispersion. Heuristically it was divided in highly dispersed (κ < 1), mildly dispersed (1 ≤ κ < 2.5) and negligibly dispersed (κ ≥ 2.5) fibres. Coincidentally, this division enclosed specific areas in the OC (red, green and blue ROIs). **(B)** After division, the cumulated data were binned irregularly as a function of the estimated voxel-wise angular orientation ( $\theta_{\vec{\mu }}$ ) per κ range (orange bars), to avoid a possible effect size bias caused by its non-uniform distribution (blue bars). The first angular irregular bin or angular offset $\theta_{0}$ was obtained and showed to be κ range dependent (Supplementary Table S1, section 5).

To compare the *α*- and *β*-parameters between datasets as a function of fibre dispersion (κ) and $\theta_{\vec{\mu }}$ , the fitted parameters were binned and averaged for the *ex vivo* cumulated data (section 3.1.5) and for the *in silico* data. The binning on the fitted parameters was performed to ensure: (1) a reduced effect size bias in the *ex vivo* cumulated data, given the unequal number of voxels at specific $\theta_{\vec{\mu }}$ and κ (Figure 5); and (2) a better comparison between *in silico* and *ex vivo* data.

In the binning process, both datasets were distributed first as a function of κ, and later as a function of $\theta_{\vec{\mu }}$ . The first distribution was performed to ensure a similar degree of fibre dispersion as observed in Figure 4B and in the work of Fritz et al. (2020). For that, three different fibre dispersion ranges were defined as a function of κ: κ < 1 for the highly dispersed fibres, 1 ≤ κ < 2.5 for the mildly dispersed fibres, and κ ≥ 2.5 for the negligibly dispersed fibres. Coincidentally, these fibre dispersion ranges depicted specific areas in the OC (Figure 5A). However, the *in silico* data required two extra averages on the fitted parameters to bin it as a function of the different fibre dispersion ranges: first, across the 5,000 replicas and, second, across the κ values within each fibre-dispersion range. The average across κ was performed in such a way that it resembled the frequency distribution of κ observed in the *ex vivo* cumulated data (for more detail, see Supplementary material, section 5). After separating the fitted parameters per fibre dispersion range for both datasets, the data were irregularly binned as a function of $\theta_{\vec{\mu }}$ bins per defined κ range.

The irregular $\theta_{\vec{\mu }}$ bins were introduced to avoid a bias due to the uneven distribution of voxels with azimuthal orientations across the 16 angular measurements (Figure 5B, blue bars). To determine the irregular $\theta_{\vec{\mu }}$ bin sizes, a cumulated $\theta_{\vec{\mu }}$ distribution of voxels was estimated and divided into 20 equally populated bins (Figure 5B, orange bars). The mean of the first angular irregular bin was defined as the angular offset $\theta_{0}$ . The range of $\theta_{\vec{\mu }}$ values contained in each irregular bin and the $\theta_{0}$ values are shown in Supplementary Table S1, section 5.

After binning, the average and standard deviation (sd) of the α- and β-parameters between datasets was calculated per irregular $\theta_{\vec{\mu }}$ bin in the *ex vivo* cumulated data. For the *in silico* data, the average and sd of the same parameters were obtained by weighting the distribution of $\theta_{\vec{\mu }}$ in each bin in a similar way to that seen in the irregular bins in the *ex vivo* cumulated data (for more detail, see Supplementary material, section 5).

#### 3.3.2. Quantitative analysis

Four different analyses were performed in order to study: (1) the effect of g-ratio and fibre dispersion, via κ, on the estimated angular-independent *β*_1_ parameter in M2, (2) the microstructural interpretability of *β*_1_ via the deviation between fitted *β*_1_ and its predicted counterparts from M2 (*β*_1,nm_, Eq. 3) and from the heuristic expression (*β*_1,m_, Eq. 4), (3) the possibility of calculating the MWF (Eq. 5) from the fitted *β*_1_ using the heuristic expression *β*_1,m_, and (4) the effect of TE, via the different meGRE subsets, on the performance of M2. The last analysis was divided into two parts, testing: (A) its capability to reduce the orientation dependence in *β*_1_ (and thus be a valid proxy for R_2,iso_*), and (B) if M2 can be better explained by the different meGRE subsets than M1. Using the simulation framework, the validity of M2 and its derived microstructural parameters were tested based on analyses 1, 2 and 4. While both datasets were used for the first and fourth analyses, only the *in silico* data were used for the second analysis while *ex vivo* data were only used for the third analysis.

##### 3.3.2.1. First analysis: ability of M2 to obtain the angular-independent β_1_ parameter for varying g-ratio and fibre dispersion values

For the first analysis, the ability of M2 to estimate an orientation-independent effective transverse relaxation rate, R_2,iso_*, via the *β*_1_ parameter was assessed. Since R_2,iso_* by definition is the angular independent part of R_2_ * and according to the HCFM should be given by *β*_1_ parameter at $\theta_{\vec{\mu }}=0\equiv \theta_{0}$ , we assessed the residual $\theta_{\vec{\mu }}$ dependence of the *β*_1_ parameter with respect to $\theta_{0}\;$ and compared it with its counterpart for *α*_1_, i.e., the proxy for the $\theta_{\vec{\mu }}$ dependent R_2_*.

For this, we first calculated the $\theta_{\vec{\mu }}$ dependence of each parameter with respect to $\theta_{0}\;$ using the normalised-root-mean-squared deviation (nRMSD, in %):


(8) $$\;nRMSD\left(\gamma \left(\kappa_{j}\right)\right)=\frac{\sqrt{\sum_{l=1}^{N-1}\frac{{\left(\gamma \left(\kappa_{j},\theta_{0}\right)-\gamma \left(\kappa_{j},\theta_{l}\right)\right)}^{2}}{N-1}\;}}{\gamma \left(\kappa_{j},\theta_{0}\right)}\cdot 100\%,$$

where $\theta_{0}$ varied slightly for each κ range (sub-index j) but was close to zero (see Supplementary Table S1) with 𝛾 ∈ {𝛼_1_, 𝛽_1_}.

To compare the nRMSD of each parameter, we calculated the difference between them, ΔnRMSD, as:

(9) $$\Delta nRMSD\left(\kappa_{j}\right)=nRMSD\left(\beta_{1}\left(\kappa_{j}\right)\right)-nRMSD\left(\alpha_{1}\left(\kappa_{j}\right)\right)$$

in percentage-points (%-points). If the ΔnRMSD is positive or higher than 0%-points, this implies that the $\theta_{\vec{\mu }}$ dependency of *β*_1_ is similar or higher, in magnitude, to *α*_1_. The latter says therefore that M2 failed in estimating an angular-independent parameter from R_2_*. A negative ΔnRMSD in turn implies that the $\theta_{\vec{\mu }}$ independence of $\beta_{1}\left(\kappa_{j}\right)$ has been reduced. A perfect orientation independence is achieved if $nRMSD\left(\beta_{1}\left(\kappa_{j}\right)\right)=0$ and, consequently, $\Delta nRMSD\left(\kappa_{j}\right)=-nRMSD\left(\alpha_{1}\left(\kappa_{j}\right)\right)$ .

##### 3.3.2.2. Second analysis: assessment of the microstructural interpretability of β_1_

For the second analysis, the microstructural interpretation of *β*_1_ was quantitatively assessed by comparing the relative difference (ε) between estimated *β*_1_ at the angular orientation $\theta_{\vec{\mu }}$ for the fitted *in silico* data ( $\beta_{1}\left(\theta_{\vec{\mu }}\right)$ ) and the predicted *β*_1_ ( $\beta_{1,p}$ ) using M2 or the heuristic expression:

(10) $$\epsilon {\left(\theta_{\vec{\mu }},\kappa_{j}\right)}_{p}=\left(1-\frac{\beta_{1}\left(\theta_{\vec{\mu }},\kappa_{j}\right)}{\beta_{1,p}}\right)\cdot 100\%,$$

where $\beta_{1,p}\in \left\{\beta_{1,nm},\beta_{1,m}\right\}$ were defined in Eqs 5, 6, respectively. Additionally, the mean $\epsilon {\left(\theta_{\vec{\mu }},\kappa_{j}\right)}_{p}$ across angles was calculated as $\langle \epsilon {\left(\kappa_{j}\right)}_{p}\rangle \equiv \frac{1}{N}\overset{N}{\underset{l=1}{\sum }}\epsilon {\left(\theta_{l},\kappa_{j}\right)}_{p}$ .

##### 3.3.2.3. Third analysis: myelin water fraction and g-ratio estimation from ex vivo data using the heuristic expression of R_2,iso_* via β_1,m_

For the third analysis, the MWF was estimated from the fitted *β*_1_ in *ex vivo* data using the analytical expression for *β*_1,m_ (Eq. 6). For that, we used the two sets of R_2_ values for the non-myelinated (R_2N_) and myelinated (R_2M_) compartments reported in Table 1. Only the *ex vivo* R_2_ values were reported in this section, while the *in vivo* R_2_ values were reported in Supplementary material, section 7.2.3.

**Table 1:** Microstructural parameters used to generate the *in silico* data.

Parameter	Value	Reference
Anisotropic and isotropic susceptibilities (χ_A_ and χ_I_)	−0.1 ppm	Wharton and Bowtell (2013)
Exchange (E)	0.02 ppm	Wharton and Bowtell (2013)
Proton density intra-and extra- axonal compartments (ρ_A_ and ρ_E_)^*^	5,000 a. u.	Wharton and Bowtell (2013)
Larmor frequency at 7 T (ω_0_)	1.873 ∙ 10^6^ rad/ms	–
Fibre volume fraction (FVF)	0.5 n. u.	Wharton and Bowtell (2013)
Proton density myelin compartment (ρ_M_)*	3,500 a. u.	Wharton and Bowtell (2013)
R_2_ intra-and extra- axonal compartments (R_2A_ = R_2E_ = R_2N_)	18.53 s^−1^ (*ex vivo*) 27.8 s^−1^ (*in vivo*)	Dula et al. (2010) Wharton and Bowtell (2013)
R_2_ myelin compartment (R_2M_)	75.41 s^−1^ (*ex vivo*) 125 s^−1^ (*in vivo*)	Dula et al. (2010) Wharton and Bowtell (2013)
Angular orientation ( $\theta_{\vec{\mu }}$ )	2°:2°:90°	–
Index of fibre dispersion (κ)	0.001:0.1:6.0	–
g-ratio	0.66, 0.73, 0.8	Emmenegger et al. (2021) only for 0.66 and Wharton and Bowtell (2013) for 0.8.^**^
Time (i.e., echo time)	3.25:3.25:53.5 ms	–

The last four parameters are the parameter or simulation space. ^*^Proton densities were scaled by a factor of 5,000 but they kept the same proton density proportion between the non-myelinated and myelinated compartments (1:0.7). ^**^ The mean g-ratio value of 0.73 was arbitrarily defined.

##### 3.3.2.4. Fourth analysis: the effect of echo time ranges on the performance of M2

In the fourth analysis, the performance of M2 was tested in two sub-analyses when using the meGRE datasets with different TE ranges (see section 3.3.1).

###### 3.3.2.4.1. First sub-analysis: assessing the residual $\theta_{\vec{\mu }}$ dependence in β_1_ for meGRE subsets with different maximum echo times

For the first sub-analysis, the orientation dependence of *β*_1_ was assessed for the different meGRE subsets from the *ex vivo* dataset and the *in silico* data for variable g-ratio. For that, *α*_1_ and *β*_1_ from M1 and M2 were compared once again as in the first analysis and the ΔnRMSD was calculated to assess the residual $\theta_{\vec{\mu }}$ dependence of *β*_1_ in comparison to the $\theta_{\vec{\mu }}$ dependence of *α*_1._

###### 3.3.2.4.2. Second sub-analysis: assessing if M2 is better explained by the data using meGRE subsets with different maximum echo times

For the second sub-analysis, the weighted-corrected Akaike Information Criterion (wAICc, Eq. 12) was introduced [more details can be found in Supplementary Equation S30, section 6 and Burnham et al. (2011)]. According to Burnham et al. (2011), the wAICc can be used to assess whether a given model (here M2) is better explained [or “supported” as introduced in Burnham et al. (2011)] by the data than a set of other models (here M1). In this work, we used the AICc (i.e., Akaike Information Criterion, AIC, with a correction for small sample sizes) instead of the AIC or the Bayesian Information Criterion (BIC) to better account for the small sample size in comparison to the number of model’s parameters. Note that to use the AIC the ratio between the sample size and the number of parameters (n/k) should be above 40 (Burnham and Anderson, 2002) and this condition was not always fulfilled in our data.

The wAICc for M2 is defined by:

(11) $$\mathrm{wAICc}=\frac{1}{\left(1+\exp\left(-0.5\Delta \mathrm{AIC}\right)\right)},$$

where ΔAICc in Eq. 12 is the difference of the AICc for models M1 and M2:


(12) ΔAICc=AICc(M1)−AICc(M2)

The AICc and wAICc were estimated per voxel (for *ex vivo*) and replica (for *in silico*) from the previous analysis using the sum-of-squares error (SSE) from the fitting of each model (see Supplementary Equation S32, section 6) and for each of the three meGRE subsets. Note that the AICc and wAICc were estimated only *ex vivo* and *in silico* data with negligible fibre dispersion (κ ≥ 2.5). Then, the averaged wAICc as well as its standard deviation (sd) were calculated. In this work, we interpreted the range of possible wAICc values in a more conservative manner. Hereby, we mainly focused on the case AICc(M1) > AICc(M2) (Eq. 12), where the resulting wAICc (Eq. 11) is greater than 0.5: a wAICc >0.73 implies that M2 is better explained by the meGRE data than M1, and a wAICc between 0.5 and 0.73 implies that M2 and M1 are ambiguously explained by the data but M2 is still preferred. For the case of AICc(M1) ≤ AICc(M2), where wAICc ≤0.5, M2 was not explained by the data as compared to M1. More details regarding the calculations as well as the threshold of 0.73 can be found in the Supplementary material, section 6. Note that we were only reporting the average wAICc, thus the wAICc for some voxels (for *ex vivo*) or replicas (for *in silico*) might belong to a different range than the average wAICc, which can be observed by the estimated sd wAICc.

In the following sections, the dependence of the parameters under study, i.e., nRMSD( $\alpha \left(\kappa_{j}\right)$ ), nRMSD( $\beta \left(\kappa_{j}\right)$ ), ΔnRMSD ( $\kappa_{j}$ ), *α*_1_( $\theta_{\vec{\mu }},\kappa_{j}$ ), *β*_1_( $\theta_{\vec{\mu }},\kappa_{j}$ ) (Eqs 8, 9), $\epsilon {\left(\theta_{\vec{\mu }},\kappa_{j}\right)}_{p}$ (Eq. 10) and $\epsilon {\left(\kappa_{j}\right)}_{p}$ , to $\theta_{\vec{\mu }}$ and κ were simplified for readability purposes. Therefore, these parameters will be hereafter nRMSD(*α*_1_), nRMSD(*β*_1_), ΔnRMSD, *α*_1_, *β*_1_, $\epsilon_{p}$ and $\epsilon_{p}$ , respectively.

## 4. Results

### 4.1. First analysis: ability of M2 to obtain the angular-independent *β*_1_ parameter for varying g-ratio and fibre dispersion values

**Figure 6:**
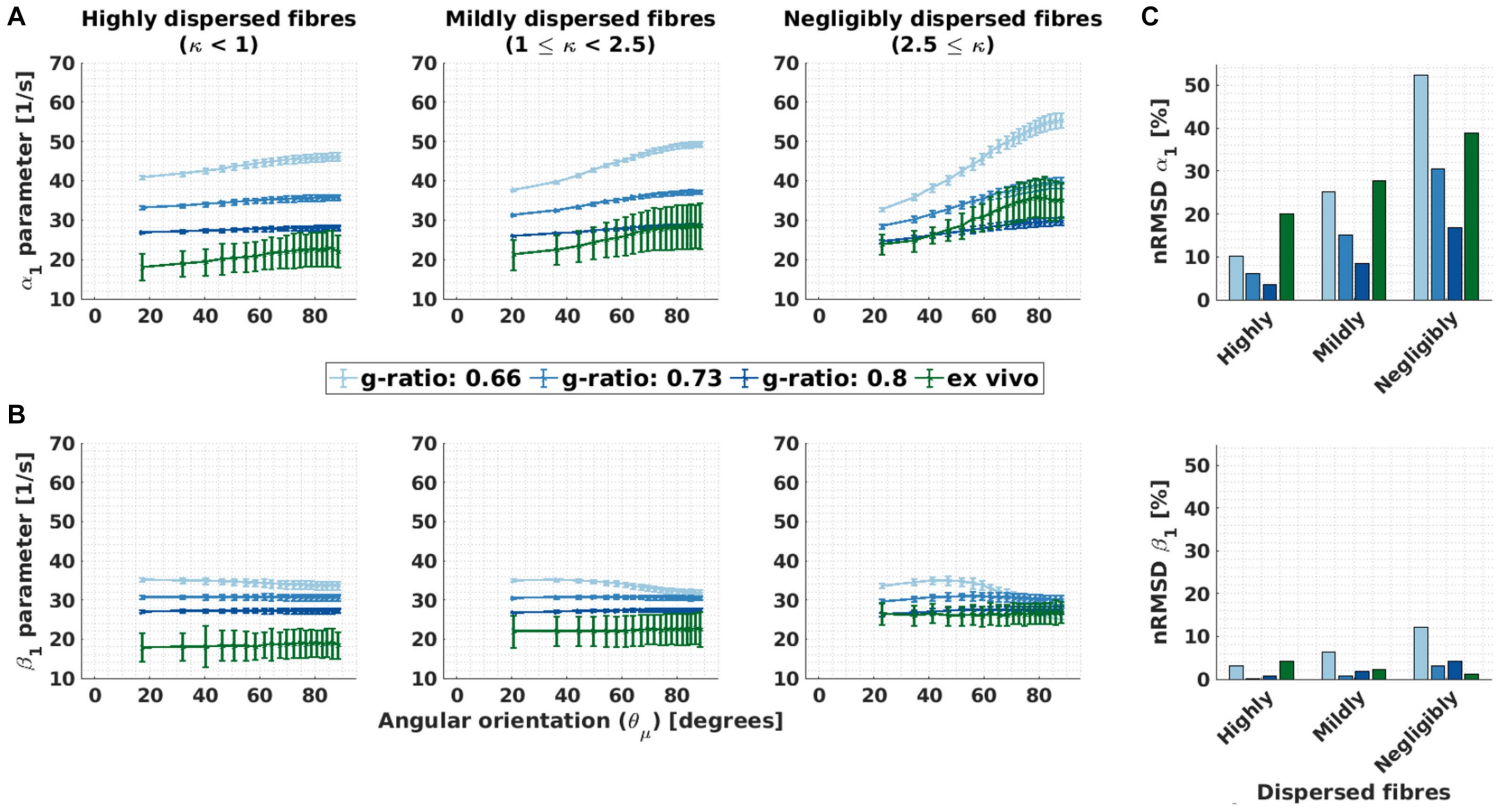
Orientation dependence of linear model parameters (α_1_ and β_1_) for varying g-ratio and fibre dispersion values. **(A,B)** Depicted is the α_1_ parameter of M1 (proxy for R_2_*) and β_1_ parameter of M2 (proxy for the isotropic part of R_2_*) as a function of the angle between the main magnetic field and the fibre orientation ( $\theta_{\vec{\mu }}$ ) for different fibre dispersion and g-ratio values. The different columns depict different dispersion regimes: highly dispersed (κ < 1, first column), mildly dispersed (1 ≤ κ < 2.5, second column) and negligibly dispersed (κ ≥ 2.5, third column) fibres. Note that the smallest angle ( $\theta_{0}$ ) varied across dispersion regimes: 17.3° (κ < 1), 20.4° (1 ≤ κ < 2.5) and 22.9° (2.5 ≤ κ). This was caused by the irregular binning (see section 3.1.4) **(C)** Depicted is the normalised root-mean-squared deviation (nRMSD, Eq. 11 in %) of the α_1_ parameter of M1 (proxy for R_2_*) and β_1_ parameter of M2 (proxy for the isotropic part of R_2_*) for different fibre dispersion and g-ratio values. Across the entire figure, the distinct colours (blue and green curves and bars) distinguish between *in silico* data with variable g-ratios (increasing blue hue with increasing g-ratio) and *ex vivo* data (olive curve).

Figure 6 shows the performance of M2 when estimating R_2,iso_* via *β*_1_ for variable g-ratio and fibre dispersion. To visualise this, we compared the $\theta_{\vec{\mu }}$ dependence of *α*_1_ from M1 to the residual $\theta_{\vec{\mu }}$ dependence of *β*_1_ from M2 (Figures 6A,B). Both $\theta_{\vec{\mu }}$ dependencies were quantified in Figure 6C using their respective nRMSD (Eq. 8). The results are from the analysis performed on the *ex vivo* and *in silico* data. The *in silico* data was generated using the *ex vivo* compartmental R_2_ values (the corresponding results for the *in vivo* compartmental R_2_ values are presented in Supplementary Figure S4).

The ability of M2 to reduce the $\theta_{\vec{\mu }}$ dependency of *β*_1_ varied with g-ratio and fibre dispersion. The $\theta_{\vec{\mu }}$ dependency of *α*_1_ (and residual $\theta_{\vec{\mu }}$ dependency of *β*_1_) was also strongly influenced by g-ratio and fibre dispersion: smaller g-ratio values and reduced fibre dispersion increased the $\theta_{\vec{\mu }}$ dependency of *α*_1_ and (the residual $\theta_{\vec{\mu }}$ dependency) of *β*_1_ (Figures 6A,B, respectively).

The fibre dispersion affected the performance of M2 the same between *in silico* and *ex vivo* datasets (Figure 6C). In both datasets, the improvement is largest for negligible dispersion (starting from ΔnRMSD = −12.0%-points for the *in silico* data with a g-ratio of 0.8 and ΔnRMSD = −37.4%-points for the *ex vivo* data). For the *ex vivo* data, the nRMSD(*β*_1_) was the lowest for the negligibly dispersed fibres (nRMSD(*β*_1_): 1.3% at κ ≥ 2.5). For the *in silico* data, the nRMSD(*β*_1_) was the lowest for the highly dispersed fibres and for a g-ratio of 0.73 (nRMSD(*β*_1_): 0.1%), and it increased with decreasing fibre dispersion (nRMSD(*β*_1_) up to 2.7%). For the g-ratios of 0.66 and 0.8, the nRMSD(*β*_1_) was higher but still below 12%.

The $\theta_{\vec{\mu }}$ dependence of *α*_1_ on fibre dispersion was the same between *in silico* and *ex vivo* datasets (Figure 6C, top): the lower the dispersion the higher the nRMSD(*α*_1_). The $\theta_{\vec{\mu }}$ dependence of *α*_1_ increased as the g-ratio decreased.

### 4.2. Second analysis: assessment of the microstructural interpretability of *β*_1_

**Figure 7:**
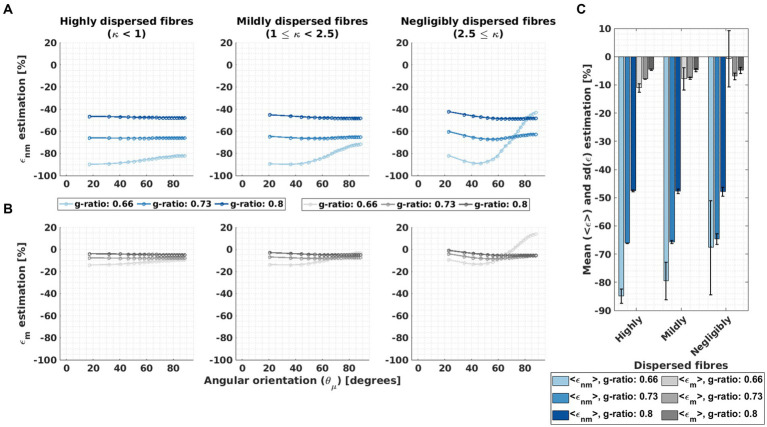
Assessment of the microstructural interpretability of β_1_ by the deviation between fitted and biophysically predicted β_1_. **(A–B)** The relative difference (ε, Equation 10) was calculated between the fitted β_1_ to the *in silico* data and two biophysically-modelled expressions for β_1_ based on the HCFM. The two expressions for β_1_ values were calculated from the original expression for M2, β_1,nm_ (Equation 3, resulting in ε_nm_, A) and the heuristic expression, β_1,m_ (Equation 4, resulting in ε_m_, B). This was calculated per g-ratio and fibre dispersion. **(C)** The corresponding mean, <ε>, and standard deviation, sd(ε), of the relative differences across the angular orientations ( $\theta_{\vec{\mu }}$ ) were estimated. The hue intensity coding represents increasing g-ratio value for both error estimations.

Figures 7A,B report the angular-orientation ( $\theta_{\vec{\mu }}$ ) dependent relative differences ( $\epsilon_{nm}$ and $\epsilon_{m}$ , Eq. 10) between the fitted *β*_1_ from the *in silico* data and its predicted counterparts using M2 (Eq. 3) and the heuristic expression (Eq. 4). Figure 7C shows the mean and standard deviation of $\epsilon_{nm}$ and $\epsilon_{m}$ across angles for *ex vivo* compartmental R_2_ values (the corresponding results for the *in vivo* R_2_ values are presented in Supplementary Figure S5).

$\epsilon_{nm}$ was large, between −100% and − 40%, and varied strongly with g-ratio and fibre dispersion. $\epsilon_{nm}$ showed the largest $\theta_{\vec{\mu }}$ dependence where the largest deviation was observed (i.e., for the g-ratio of 0.66 and the lowest fibre dispersion, Figure 7A). $\epsilon_{m}$ was always smaller than $\epsilon_{nm}$ and showed a smaller $\theta_{\vec{\mu }}$ dependence across all the studied fibre dispersions and g-ratios. It varied between −20 and 20% and had the largest values and variation for the smallest g-ratio and negligibly fibre dispersion. For the average across angles, we found that negligibly dispersed fibres showed the smallest $\epsilon_{nm}$ and $\epsilon_{m}$ per g-ratio.

The mean across angles for $\epsilon_{nm}$ , $\langle \epsilon_{nm}\rangle$ , was up to −85% whereas the mean across angles for $\epsilon_{m}$ , $\langle \epsilon_{m}\rangle$ , was only up to −12% (Figure 7C). On average across all g-ratios and fibre dispersion arrangements, $\langle \epsilon_{nm}\rangle$ was approximately 8 to 9 times larger than $\langle \epsilon_{m}\rangle$ . Both relative mean differences became more negative with increasing g-ratio and decreasing fibre dispersion. The $\langle \epsilon_{m}\rangle$ for the negligibly dispersed fibres at g-ratio 0.66 was close to −2% but accompanied by a large standard deviation across $\theta_{\vec{\mu }}$ due to the strong $\theta_{\vec{\mu }}$ -dependency of the corresponding fitted $\beta_{1}$ parameters. For both $\epsilon_{nm}$ and $\epsilon_{m}$ , the variability (Figure 7C) across different $\theta_{\vec{\mu }}$ values, $sd\left(\epsilon_{nm}\right)$ and $sd\left(\epsilon_{m}\right)$ respectively, was highest when the fibre dispersion and g-ratio were lowest.

### 4.3. Third analysis: myelin water fraction estimation from *ex vivo* data using the heuristic expression of R_2,iso_* via *β*_1,m_

**Figure 8:**
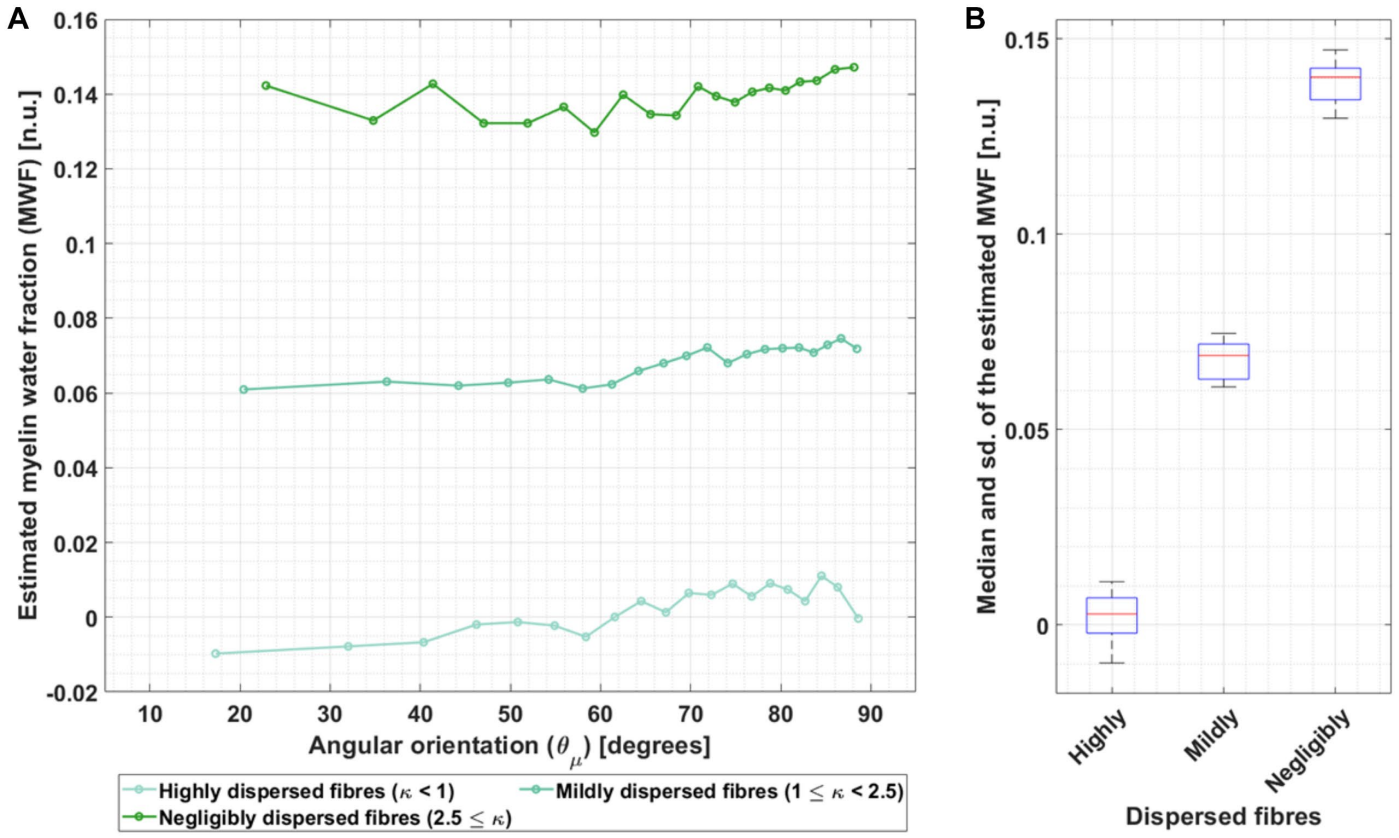
Dependence of the MWF estimation on angular orientation for three different fibre dispersion ranges in *ex vivo* data. **(A)** The MWF was estimated by using the heuristic analytical expression of β_1_ (β_1,m_, Eq. 4) and the fitted β_1_ for the *ex vivo* data using the compartmental R_2_ values from Dula et al. (2010) (hues of green) in Table 1. This calculation was performed per angle ( $\theta_{\vec{\mu }}$ ) and for the three different fibre dispersion ranges: highly dispersed, mildly dispersed and negligibly dispersed. The increasing green hue represents decreasing fibre dispersion. **(B)** The corresponding median and standard deviation (sd) were estimated across $\theta_{\vec{\mu }}$ per fibre dispersion range.

Figure 8 reports the MWF estimated from the *ex vivo* data by inverting the heuristic expression for *β*_1,m_ (Eq. 6), using the *ex vivo* compartmental R_2_ values (the corresponding results for the *in vivo* R_2_ values are presented in Supplementary Figure S6). Figure 8A shows the estimated MWF as a function of $\theta_{\vec{\mu }}$ while Figure 8B shows the median and standard deviation (sd) of the estimated MWF across $\theta_{\vec{\mu }}$ .

The estimated MWF was larger with decreasing fibre dispersion (Figure 8A). Moreover, there was a trend towards larger estimated MWF for larger $\theta_{\vec{\mu }}$ . Across $\theta_{\vec{\mu }}$ , the estimated median *ex vivo* MWF was 0.14 for fibres with negligible dispersion but moved towards to even lower and unrealistically small values (MWF: 0.069) for dispersed fibres (Figure 8B). The standard deviation across MWF was similar for different fibre dispersions, ranging from 0.0068 to 0.0104.

### 4.4. Fourth analysis: the effect of echo time ranges on the performance of M2

In this section, two sub-analyses were performed for *in silico* data at variable g-ratio and *ex vivo* data, both with negligibly dispersed fibres (i.e., κ ≥ 2.5), using the three meGRE subsets with different maximum echo time (TE_max_) for *ex vivo* compartmental R_2_ values (the corresponding results for the *in vivo* R_2_ values are presented in Supplementary Figures S7, S8). In the first sub-analysis, its result is depicted similarly as in Figure 6, but for different TE_max_ and κ ≥ 2.5. In the second sub-analysis, it was assessed whether M2 was better explained by the different meGRE subsets than M1 using the average wAICc of M2 (Eq. 11).

#### 4.4.1. First sub-analysis: assessing the residual $\theta_{\vec{\mu }}$ dependence in β_1_ for meGRE subsets with different maximum echo times

**Figure 9:**
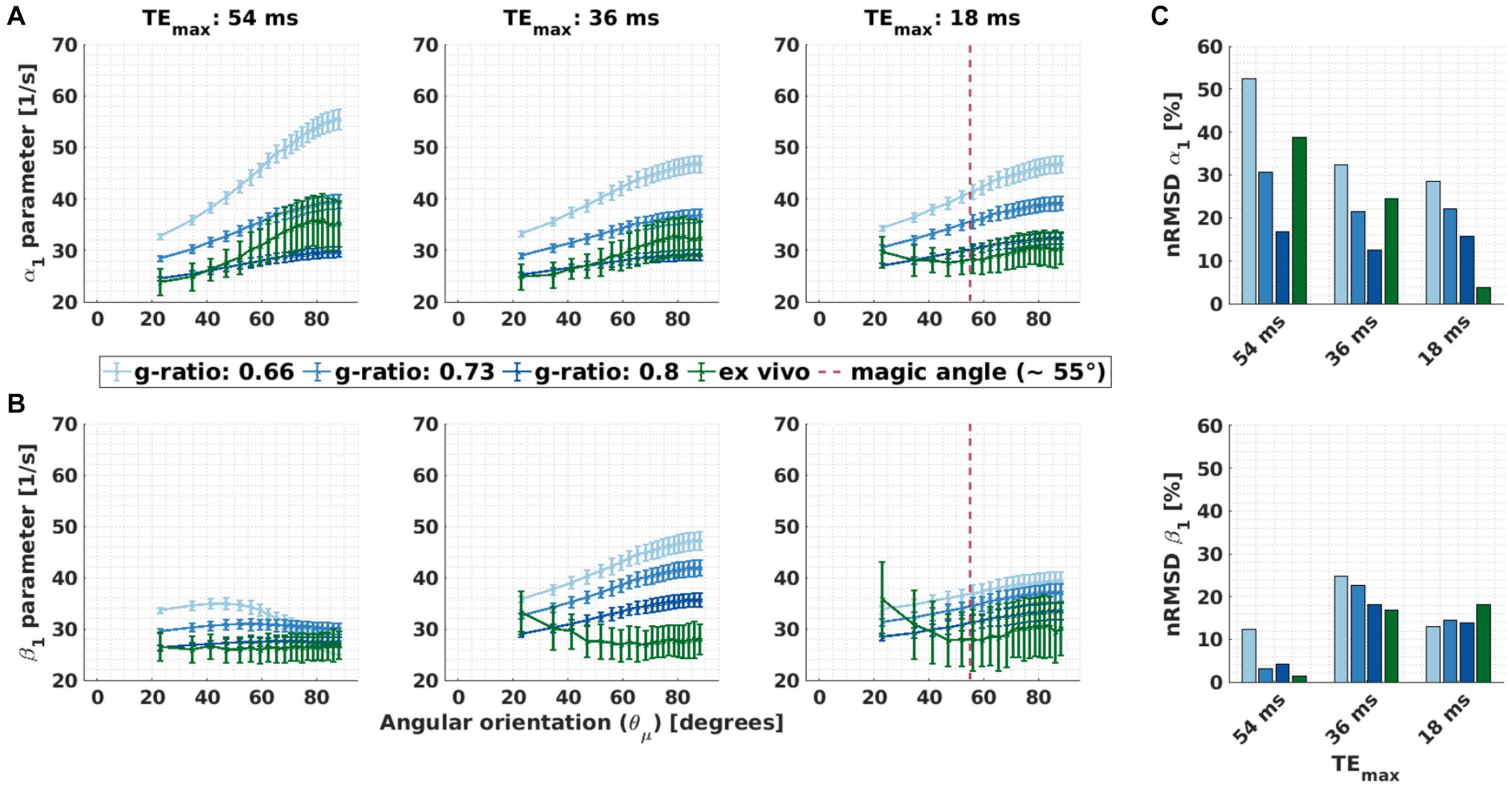
Effect of the maximal echo time, i.e., meGRE subsets with different maximum echo times, on the $\theta_{\vec{\mu }}$ dependency of α_1_ and β_1_. **(A,B)** Angular orientation ( $\theta_{\vec{\mu }}$ ) dependence of α_1_ in M1 and β_1_ in M2 for the three meGRE subsets with varying maximum TE (TE_max_: 54 ms, 36 ms and 18 ms). Two datasets are compared: *ex vivo* (green curve) and *in silico* (blue curve) data at variable g-ratios. Only datasets of the negligibly dispersed fibres (κ ≥ 2.5) are presented. The magenta vertical lines in some of the subplots indicates the magic angle ( $\theta_{\vec{\mu }}$ = 55°). **(C)** Depicted is the normalised root-mean-squared deviation (nRMSD, Eq. 8 in %) of the α_1_ parameter of M1 (proxy for R_2_*) and β_1_ parameter of M2 (proxy for the isotropic part of R_2_*) shown in **(A)** and **(B)**, respectively.

Using the meGRE subsets with smaller TE_max_ (36 ms and 18 ms), M2 was less effective across all g-ratios (Figures 9A,B, second and third column). For some microstructural parameter settings, even an increased $\theta_{\vec{\mu }}$ dependence was observed for *β*_1_ compared to *α*_1_: nRMSD(*β*_1_) went up by 5.6%-points at 36 ms (*in silico*, g-ratio: 0.8) and by 14.1%-points at 18 ms (*ex vivo*). Moreover, for the meGRE subset with the smallest TE_max_ (18 ms), an atypical $\theta_{\vec{\mu }}$ dependence of *β*_1_ (and *α*_1_) was found in the *ex vivo* data: *β*_1_ (and *α*_1_) decreased with increasing $\theta_{\vec{\mu }}$ up to approximately 55° (magic angle, dashed magenta lines in Figures 9A,B) and then slightly increased again. The $\theta_{\vec{\mu }}$ dependence up to the magic angle was not observed in the *in silico* data at any investigated meGRE subset. Moreover, the $\theta_{\vec{\mu }}$ dependence of *α*_1_ in the *ex vivo* data decreased when meGRE subsets with decreasing TE_max_ were used. This trend was mostly also observable in the *in silico* data (Figure 9A). Note that we investigated the orientation dependence of *α*_1_ and *β*_1_ also for mildly and highly dispersed fibres but did not find new trends in those datasets (data not shown).

#### 4.4.2. Second sub-analysis: assessing if M2 is better explained by the data using meGRE subsets with different maximum echo times

**Figure 10:**
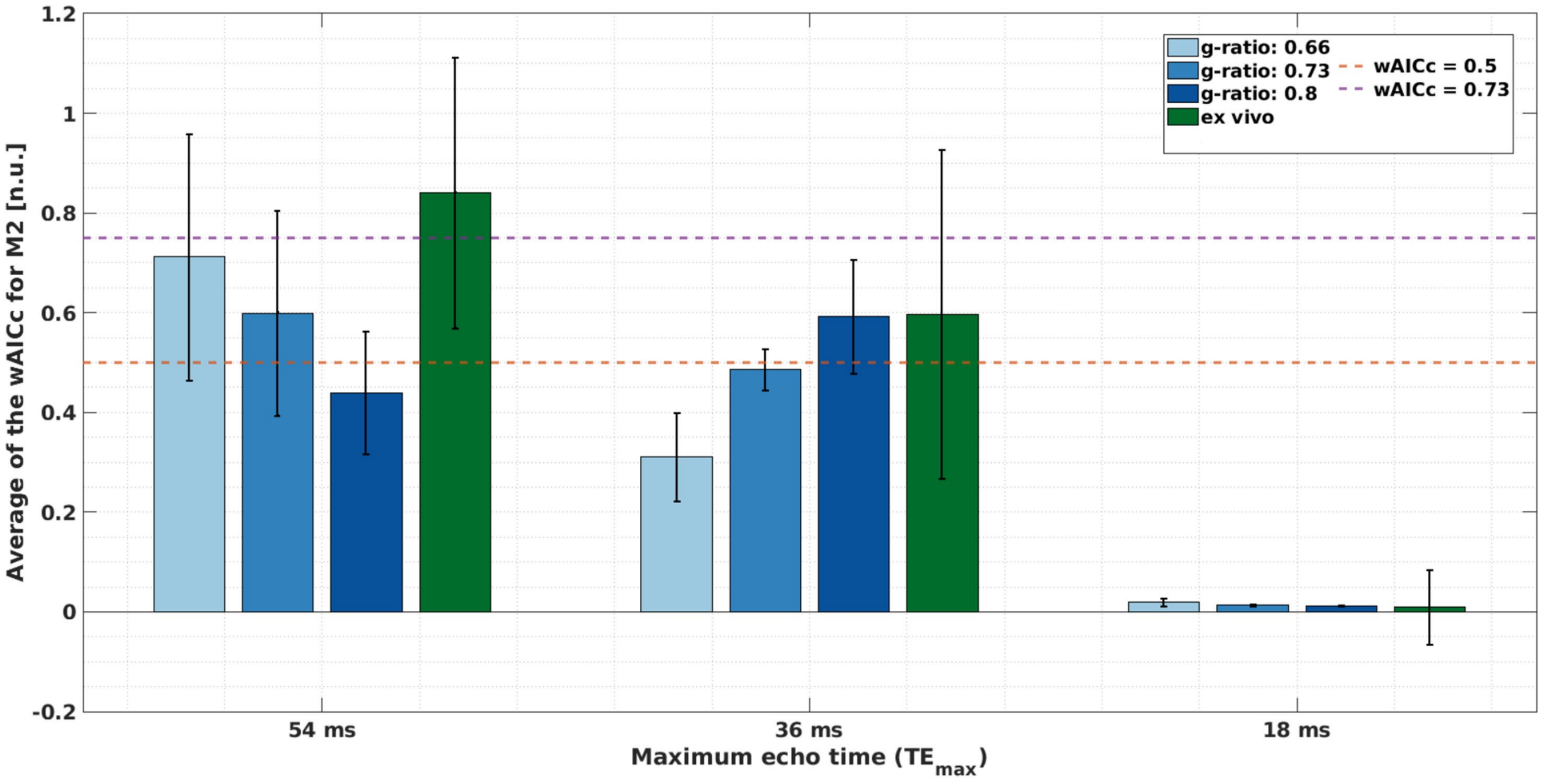
Assessing if model M2 is better explained by the meGRE signal decay than M1, quantified by the averaged wAICc for M2 (Eq. 11). This quantification was done per meGRE subsets with different maximum echo time (TE_max_) for the *in silico* data at variable g-ratios (increased blue hue in bars, higher g-ratio) with R_2_ values from Dula et al. (2010); and *ex vivo* data (green bar) for negligibly dispersed fibres (κ > 2.5). The magenta and orange lines mark the following ranges: over the magenta line (wAICc = 0.73), M2 is better explained by the data; between the magenta and orange (wAICc = 0.5) lines, there is a preference for M2 but it is ambiguous whether M2 is better explained than M1 by the data; and bellow the orange line, M2 is not better explained by the data than M1.

The average wAICc showed different trends across the different meGRE subsets with varying TE_max_ for both datasets. For the *ex vivo* data, the average wAICc decreased when meGRE subsets with smaller TE_max_ were used. Using the meGRE subsets with the largest and intermediate TE_max_ (54 and 36 ms), the average wAICc indicated that M2 was better explained than M1 by the data with wAICc values in the ranges of wAICc >0.73 (TE_max_ = 54 ms) and 0.73 > wAICc >0.5 (TE_max_ = 36 ms), respectively. Interestingly, for the *in silico* data, the average wAICc decreased as a function of g-ratio for the meGRE subset with the largest TE_max_, from wAICc: 0.71 to 0.44; but increased with increasing g-ratio for the meGRE subset with intermediate TE_max_, from wAICc: 0.31 to 0.59. However, none of the highest wAICc overpassed the threshold of 0.73. Note that the large standard deviation of the reported wAICc per dataset indicates that the results are only valid on average whereas the wAICc for single voxels (*ex vivo* data) or replicas (*in silico* data) can be outside the reported ranges (see Figure 10).

## 5. Discussion

This work quantitatively evaluated the performance of the log-quadratic model (M2) for estimating the orientation-independent part of R_2_* (R_2,iso_*) via its linear parameter, *β*_1_, using a single-orientation multi-echo GRE (meGRE) measurement in simulations and in a human optic chiasm. We found that M2 can estimate R_2,iso_* via *β*_1_ when using meGRE with long maximum echo time (TE_max_ ≈ 54 ms) for all investigated fibre dispersion and g-ratios. Our simulation results show that the proposed heuristic expression for *β*_1_ better explained the fitted *β*_1_ for *ex vivo* compartmental R_2_ values than the M2-based prediction. Using this heuristic model, we estimated realistic MWF values from *β*_1_ fitted to the *ex vivo* data. However, we found that its validity depends on the choice of compartmental R_2_-values and we found that the heuristic model cannot be used for tissue with dispersed fibres. We created an openly available simulation framework to test the validity of the heuristic expression for different microstructural arrangements. We found that M2 cannot reduce the orientation dependence of *β*_1_, and therefore cannot be used as a proxy of R_2,iso_* when the meGRE subsets with shorter maximum echo times were used (TE_max_ ≈ 36 ms or 18 ms). For the meGRE subset with the shortest TE_max_ of 18 ms, we found that the orientation-dependence of the classical R_2_* showed the highest deviation between *ex vivo* and *in silico* data for angles below the magic angle (55°), indicating that, at short echo times, the mechanism for the orientation-dependence of R_2_* is not captured by our HCFM-based simulation.

### 5.1. Ability of M2 to estimate the angular independent *β*_1_ for varying g-ratio and fibre dispersion values

Our results show that M2 has the potential to estimate R_2,iso_* from a single-orientation meGRE via *β*_1_ for the *ex vivo* data of an optic chiasm tissue sample and the *in silico* data. We found that the performance of M2, assessed by the residual $\theta_{\vec{\mu }}$ dependence of *β*_1_, varied for different g-ratios and fibre dispersions (Figure 5 and Supplementary Figure S4). For the *ex vivo* compartmental R_2_ values (Figure 5), the residual $\theta_{\vec{\mu }}$ dependence of *β*_1_ was always less than 12% even if the $\theta_{\vec{\mu }}$ dependence of the original R_2_* (using the *α*_1_ parameter of M1) was up to 50%. For the *in vivo* compartmental R_2_ values (Supplementary Figure S4), the residual $\theta_{\vec{\mu }}$ dependence of *β*_1_ was always less than 20%. The comparison of the performance of M2 for different compartmental R_2_ values indicates that the performance of M2 might vary for tissue with different microstructural tissue properties such as the compartmental R_2_ values or the fibre volume fraction.

### 5.2. Assessment of the microstructural interpretability of *β*_1_

As hypothesised in the introduction, the fitted *β*_1_ parameter is an unsuitable proxy for estimating microscopic tissue parameters via the dependency of M2 on the biophysical HCFM (Eq. 3). Using the *ex vivo* compartmental R_2_ values to generate the *in silico* data, we obtained an error of up to −70% for the fibres with negligible dispersion (Figure 7C) between the fitted *β*_1_ and the *β*_1_ predicted using the biophysical relation in M2 (Eq. 3). With the proposed heuristic expression for *β*_1_ (Eq. 4), the relative error was reduced by a factor of about 10 and more for fibres with negligible dispersion (e.g., from −65% to −6% for a g-ratio of 0.73, Figure 7C), indicating that this expression is better suited for the biophysical interpretation of *β*_1_ than the M2-based expression. However, we also found that the heuristic expression is not valid for all microstructure parameters, e.g., for *in vivo* compartmental R_2_ values the error switched signed, e.g., it changed from −35 to 20% for a g-ratio of 0.73 and negligible fibre dispersion (Supplementary Figure S6). This shows that the validity of the new heuristic expression for *β*_1_ as a sum of the relaxation rates of the myelin and non-myelin water pools weighted by their signal fractions is constrained to a specific range of relaxation rate values.

In this manuscript, we provide a simulation framework that allows to test whether for a given set of microscopic parameters the validity of the heuristic expression is given.

Note that neither the proposed heuristic correction nor the previous M2-based expression account for the effect of fibre dispersion which might explain why the accuracy of the predictions decreased with increasing fibre dispersion (Figure 7). While the influence of fibre dispersion has been successfully incorporated into M2 in another study (Fritz et al., 2020), it remains an open task for future studies to also do this for the heuristic expression of *β*_1_.

### 5.3. Myelin water fraction estimation from *ex vivo* data using the heuristic expression for *β*_1_

Under the condition that M2 estimates an orientation-independent *β*_1_ and that the heuristic expression of *β*_1_ provides a valid biophysical interpretation, the myelin water fraction (MWF) can be estimated from the fitted *β*_1_ (Eq. 6). When using the *ex vivo* compartmental R_2_ values, we found a median (across orientation) MWF of 0.14 for fibres with negligible dispersion (Figure 8B), which is congruent with the mean value reported in white matter of 0.10 (Uddin et al., 2019). In the Supplementary materials section 7.2.3, we exemplified what happens if the MWF is calculated for a set of microscopic parameters for which the heuristic expression is invalid. We found that the resulting MWF is negative and thus implausible. As such, the estimation of the MWF through *β*_1_ seems a less effective method than existing MWF estimation approaches but might still be useful to estimate the MWF if magnitude-only meGRE data with a single head orientation are available.

### 5.4. The effect of echo time on the performance of M2

Our findings revealed that the ability of M2 to estimate *β*_1_ was reduced for meGRE subsets with shorter maximum echo time (TE_max_). This was evidenced by: (i) an increased residual orientation dependence of *β*_1_, and (ii) M2 not being better explained by the meGRE data than M1. The performance of M2 decreased when the maximum TE (TE_max_) also decreased. This was not only observed for meGRE subsets with TE_max_ values typically used for *in vivo* studies (i.e., TE_max_ = 18 ms), but also at the intermediate TE_max_ (= 36 ms). Note that these observations could also be driven by the reduced time points of the meGRE subsets at shorter TE_max_: while the meGRE subset at TE_max_ = 54 ms contained 16 time points, the meGRE subset at TE_max_ = 18 ms only contained five time points. A limited sample size or number of time points, however, is an unsolved challenge for *in vivo* application of M2 because typical *in vivo* meGRE protocols, specifically MPM protocols, use short TE_max_ (~ 18 ms) and few echo times only (~ 6–8 echoes). Therefore, future studies should aim at increasing the TE_max_ and/or the time points. This will require highly accelerated acquisitions [e.g., like in Han et al. (2014) for spin echo sequences or Kim et al. (2019) for 3D-GRE sequences] and the correction of motion artefacts (Magerkurth et al., 2011), B_0_ fluctuations due to breathing (e.g., Vannesjo et al., 2015) and susceptibility artefacts (e.g., Port and Pomper, 2000), which are particularly strong at later echo times.

Interestingly, the biggest discrepancy between *in silico* and *ex vivo* results for *β*_1_ was seen for the meGRE subset with the shortest TE_max_ value at $\theta_{\vec{\mu }}$ smaller than the magic angle (55°, Figure 9B). This is because *β*_1_ and *α*_1_ of the measured *ex vivo* data showed an atypical $\theta_{\vec{\mu }}$ dependence in this $\theta_{\vec{\mu }}$ range: they decreased as a function of increasing $\theta_{\vec{\mu }}$ up to the magic angle. A similar observation was also made by Bartels et al. (2022) for the orientation dependence of R_2_. They suggested that a mechanism that could explain a reduction in R_2_ at the magic angle would be the Magic Angle Effect in highly structured molecules like myelin sheaths (see Bydder et al., 2007). Since, in our experiment, this phenomenon would be superimposed on the orientation dependence of R_2_*, it may be particularly evident when the latter effect is negligible, i.e., at low $\theta_{\vec{\mu }}$ . Note that our finding was observed only for one tissue sample. Thus, further testing on different tissue samples is necessary to verify the generalisability of our finding.

### 5.5. Considerations

Our results indicate that the ability of M2 to estimate the orientation-independent component of R_2_* varies with echo time and strongly depends on microstructural parameters. As the space of parameters in the simulations are large, not all possible combinations could be investigated here. In future studies, we will test the performance of M2 in scenarios that map directly to *in vivo* meGRE experiments as opposed to the *ex vivo* case that was the focus of this study.

M2 can separate the orientation dependence of R_2_* leaving an orientation-independent parameter *β*_1_, but at the same time this estimated *β*_1_ cannot be predicted accurately based on the current analytical derivation of M2 (Supplementary Equations S15, S16, section 4). Future studies should aim to find a better derivation of M2 from the HCFM that does not neglect the contribution of the myelin water as well as incorporating other sources of dephasing, e.g., due to diffusion and near-field interactions. In fact, an analytical derivation without neglecting the contribution of the myelin compartment was performed in this manuscript (Supplementary material, section 4). However, this derivation is mathematically valid only for meGRE subsets with a maximal TE smaller than the T_2_ of the myelin compartment. Thus, the derived expression (Supplementary Equations S13, S14, section 4) does not hold for our simulated datasets because TE_max_ > T_2_ myelin for all meGRE subsets. This might also explain why the heuristic expression does not work for the *in vivo* compartmental R_2_ values, for which the T_2_ myelin is smaller. Nevertheless, it can be used to motivate our heuristic expression for *β*_1_ (Eq. 4) because it is the same expression as in Supplementary Equation S15b. This derivation might also be relevant for studies that are performed at lower magnetic fields, e.g., at 3 T, where the condition TE_max_ > T_2_ myelin could be fulfilled because the R_2_ from the myelinated and non-myelinated compartments are different (e.g., shorter) from the ones used in our current simulation.

Our simulations did not always show the same trend as the *ex vivo* data (e.g., Figures 6, 9) and were occasionally quantitatively different. This could be related to simplifications that were employed in our simulations and/or the underlying simplifications of the HCFM. The most important simplifications in our simulations were: First, the assumption that the R_2_ was the same for both intra- and extracellular compartments. Although, these R_2_ have been found to be different (e.g., Beaulieu et al., 1998; Assaf and Cohen, 2000; Does and Gore, 2000; Does, 2018; Veraart et al., 2018; Tax et al., 2021), we expect the differences not to play a substantial role at the short TEs that were used here [e.g., TE_max_: 54 ms < T_2_ of the extra-axonal compartment ≈ 58 ms in Tax et al. (2021)]. Second, we assumed that the signal coming from multiple dispersed hollow cylinders is a superposition of the complex signal of multiple single hollow cylinders at different orientations, neglecting the near-field interaction of the cylinders. As compared to previous studies where near-field interaction was more faithfully described in two dimensions (Xu et al., 2018; Hédouin et al., 2021), our simulation framework allowed for better control over the fibre dispersion in three dimensions via the Watson distribution parameter κ. The most important simplifications of the HCFM are: (1) neglecting the orientation dependence of R_2_ with respect to the external magnetic field (Knight et al., 2017; Birkl et al., 2021; Tax et al., 2021) and (2) the different longitudinal magnetisation of the compartments which affects the longitudinal relaxation rate (R_1_) (see, e.g., Labadie et al., 2014; Shin et al., 2019; van Gelderen and Duyn, 2019; Chan and Marques 2020; Kleban et al., 2021). While the anisotropic part of R_2_ is three times smaller than the anisotropic part of R_2_* at 3 T (Gil et al., 2016) and could explain residual orientation dependence of *β*_1_, other assumptions requires further study, for example removing the R_1_ dependence in the estimated R_2_* (Milotta et al., 2023). Nevertheless, even with all the simplifications, the HCFM-based *in silico* data described the $\theta_{\vec{\mu }}$ dependence of *α*_1_ and *β*_1_ similarly to the *ex vivo* data across all dispersion regimes when using the long maximal TE protocol.

The *ex vivo* data require further discussion. First, we investigated only one human optic chiasm tissue sample with relatively long *postmortem* interval of 48 h, which could explain parts of the differences that we found when comparing with the *in silico* dataset. Second, the coregistration of the diffusion and meGRE datasets (see section 3.1.4) might lead to image interpolation artefacts affecting the κ and $\theta_{\vec{\mu }}$ estimates. Moreover, coregistration between meGRE images at different orientations could lead to additional blurring of the data. However, these coregistration steps are necessary to ensure maximal correspondence between the same voxels across maps. We expect that the additional coregistration-related blurring will only slightly reduce the variability when binning the data (e.g., the standard deviation along R_2_* in Figure 6). Third, the Watson dispersion from the NODDI model cannot describe all existing fibre arrangements in the brain accurately, e.g., the crossing fibre arrangement. However, in the optic chiasm specimen crossing-fibre arrangements were only found in a few regions, e.g., at the crossing of the optical tract and optic nerve. Therefore, the contribution of such crossing-fibre voxels with estimated κ values in the range of highly to mildly dispersed fibres will be averaged-out with the single-fibre orientation voxels with similar κ values during the irregular binning pre-processing (section 3.3.1). However, this could result in an increasing standard deviation in the estimated *α*-parameters in the log-linear model and *β*-parameters in the log-quadratic model.

## 6. Conclusion

We showed that our recently introduced biophysical log-quadratic model (M2) of the multi-echo gradient-recall echo (meGRE) signal can estimate the fibre-angular-orientation independent part of R_2_* (R_2,iso_*) for varying g-ratio values and fibre dispersions. Thus, the estimated linear time-dependent parameter of M2, *β*_1_, provides an attractive alternative for estimating R_2,iso_* to standard methods that require multiple acquisitions with distinct positioning of the sample in the head-coil. We also showed that *β*_1_ can be used to estimate the myelin water fraction (MWF) for *ex vivo* compartmental R_2_ values using a newly proposed heuristic expression relating *β*_1_ to microstructural tissue parameters including the myelin water signal. We provide a freely available simulation framework to test the validity of the heuristic expression for varying sets of microstructural parameters. We found that the heuristic expression cannot be used for *in vivo* compartmental R_2_ values.

Importantly, we found that an angular-independent *β*_1_ (and thus R_2,iso_*) cannot be estimated with the log-quadratic model for meGRE measurements with maximum shorter echo times, that are typically used for whole-brain *in vivo* meGRE experiments. Therefore, it indicates that we need to develop new meGRE protocols with longer echo times that remain time efficient and motion robust. This could be achieved by using highly accelerated acquisitions with a higher data sampling for shorter echo times. Finally, at echo time ranges of about 18 ms, an unexpected R_2_* orientation-dependence was found in the *ex vivo* dataset at angles below the magic angle: a decrease of R_2_* for increasing angles. However, more testing is required to confirm that our finding can be generalised to other brain regions and specimens since our results are based on thorough measurements of one human optic chiasm tissue sample.

## Data availability statement

The raw data supporting the conclusions of this article will be made available by the authors, without undue reservation.

## Author contributions

FF: conceptualization, MRI data analysis, in-silico data analysis and manuscript’s writer. LM and MC: manuscript review. MA: MRI data pre-processing and acquisition. JP and AP: MRI data acquisition and protocol design. MM: *Ex-vivo* specimen preparation and containment, manuscript review. CJ: *Ex-vivo* specimen preparation and containment. TN and NW: resources and manuscript review. KP: MRI data acquisition and protocol design, manuscript review. SM: conceptualization, funding acquisition, co-writer of the manuscript and manuscript review, supervision. All authors contributed to the article and approved the submitted version.

## Funding

This work was supported by the German Research Foundation (DFG Priority Program 2041 “Computational Connectomics,” [AL 1156/2-1;GE 2967/1-1; MO 2397/5-1; MO 2249/3-1; MO 2397/5-2], by the Emmy Noether Stipend: MO 2397/4-1, MO 2397/4-2) and by the BMBF (01EW1711A and B) in the framework of ERA-NET NEURON and the Forschungszentrums Medizintechnik Hamburg (fmthh; grant 01fmthh2017). The research leading to these results has received funding from the European Research Council under the European Union’s Seventh Framework Programme (FP7/2007–2013) / ERC grant agreement n° 616,905. MFC is supported by the MRC and Spinal Research Charity through the ERA-NET Neuron joint call (MR/R000050/1). The Wellcome Centre for Human Neuroimaging is supported by core funding from the Wellcome [203,147/Z/16/Z]. The Max Planck Institute for Human Cognitive and Brain Sciences has an institutional research agreement with Siemens Healthcare. NW holds a patent on acquisition of MRI data during spoiler gradients (US 10,401,453 B2). NW was a speaker at an event organized by Siemens Healthcare and was reimbursed for the travel expenses.

## Conflict of interest

The authors declare that the research was conducted in the absence of any commercial or financial relationships that could be construed as a potential conflict of interest.

## Publisher’s note

All claims expressed in this article are solely those of the authors and do not necessarily represent those of their affiliated organizations, or those of the publisher, the editors and the reviewers. Any product that may be evaluated in this article, or claim that may be made by its manufacturer, is not guaranteed or endorsed by the publisher.

## Figures and Tables

**Table:** 

Acronyms	
Biophysical terms and model parameters	
AWF	(Intra-) Axonal water fraction
EWF	Extra-axonal water fraction
FVF	Fibre volume fraction
HCFM	Hollow cylinder fibre model
ICVF	Intra-cellular volume fraction (from NODDI)
MWF	Myelin water fraction (Eq. 6)
g_ratio_	g-ratio
Magnetic resonance imaging and sequence acronyms	
dMRI	Diffusion-weighted Magnetic Resonance Imaging
DWI	Diffusion-weighting Imaging
GRE	Gradient-recalled echo
meGRE	Multi-echo gradient-recalled echo
OC	Optic chiasm
R_2_*	Effective transverse relaxation rate
R_2,iso_*	Orientation independent or isotropic part of R_2_*
TE	Echo time
TE_max_	Maximal echo time
Hollow cylinder fibre model parameters	
S_A_	Signal of the intra-axonal compartment
S_E_	Signal of the extra-axonal compartment
S_M_	Signal of the myelin compartment
S_N_	Sum of the signals of the non-myelinated (S_A_ and S_E_) compartments
S_C_	Sum of all the signal compartments (S_A_, S_E_ and S_M_)
R_2A_	Transverse relaxation rate of the intra-axonal compartment
R_2E_	Transverse relaxation rate of the extra-axonal compartment
R_2N_	Transverse relaxation rate of the non-myelinated compartments
R_2M_	Transverse relaxation rate of the myelin compartment
ρ_A_	Proton density of the intra-axonal compartment
ρ_E_	Proton density of the extra-axonal compartment
ρ_N_	Proton density of the non-myelinated compartments
ρ_N_	Proton density of the myelin compartment
V_M_	Volume fraction of the myelin compartment
Symbols	
In silico and ex vivo data descriptors	
$\theta \vec{\mu }$	Angular orientation of the mean fibre bundle
$\theta_{0}$	First angular orientation or angular offset
$\vec{B_{0}}$	Main magnetic field
κ	Coefficient of dispersion (from Watson Distribution and NODDI)
$\vec{\mu }$	Vector of the mean fibre bundle
$\vec{x}$	Vector of the individual cylinder in the simulated in silico data
$T_{\mathrm{Diff},\mathrm{GRE}}$	Transformation matrix from dMRI to GRE images
$T_{\mathrm{GRE}:i,1}$	Transformation matrix from GRE images at the i-th angular orientation measurement to the first angular orientation measurement
Model parameters and analysis metrics	
α_0_	Intercept parameter of M1
α_1_	Slope or linear parameter of M1
β_0_	Intercept of M2
β_1_	Slope of linear parameter of M2
β_1,nm_	β_1_ ground-truth value without myelin signal contribution (Eq. 3)
β_1,m_	β_1_ ground-truth value with myelin signal contribution (Eq. 4)
β_2_	Quadrature or second order parameter of M2
ε_m_	Relative difference between fitted β_1_ and predicted β_1,nm_
ε_nm_	Relative difference between fitted β_1_ and predicted β_1,m_
AIC	Akaike Information Criterion
AICc	Akaike Information Criterion corrected
ΔAICc	Difference of Akaike Information Criteria (Equation 13)
wAICc	Weighted Akaike Information Criterion corrected (Eq. 12)
nRMSD	Normalised root-mean-squared deviation (Eq. 9)
ΔRMSD	Normalised root-mean-squared deviation difference (Eq. 10)
M1	Log-linear model (Eq. 2)
M2	Log-quadratic model (Eq. 1)

## Footnotes

^1^http://www.slicer.org

^2^http://www.fil.ion.ucl.ac.uk/spm

^3^https://github.com/quantitative-mri-and-in-vivo-histology/r2s_iso_estimation
