# Supplementary material for: Fiber-orientation independent component of R2* obtained from single-orientation MRI measurements in simulations and a post-mortem human optic chiasm
Source: Front Neurosci. 2023 Aug 25;17:1133086. doi: 10.3389/fnins.2023.1133086 (PMC10491021; doi:10.3389/fnins.2023.1133086)
Supplement: Supplementary file 1 [file Data_Sheet_1.pdf]

Supplementary material

1. Supplementary figure

Figure S1 shows eight of the sixteen angular-measured optic chiasm maps after coregistration (section 3.1.4 in main manuscript). For each measurement, the estimated voxel-wise angular ($\theta_{\vec{\mu}}$) map with the corresponding angular orientation of the external magnetic field ($\theta_{\overrightarrow{B_0}}$) are shown. The $\theta_{\overrightarrow{B_0}}$ was estimated as $\theta_{\overrightarrow{B_0}} = acos\left(\overrightarrow{B_0}(\theta_i) \cdot \overrightarrow{B_0}(\theta_0)\right)$. Middle and bottom rows also show the estimated R_2* via α_1 in M1 (Equation 2) and $R_{2,iso}$* via β_1 in M2 (Equation 1).

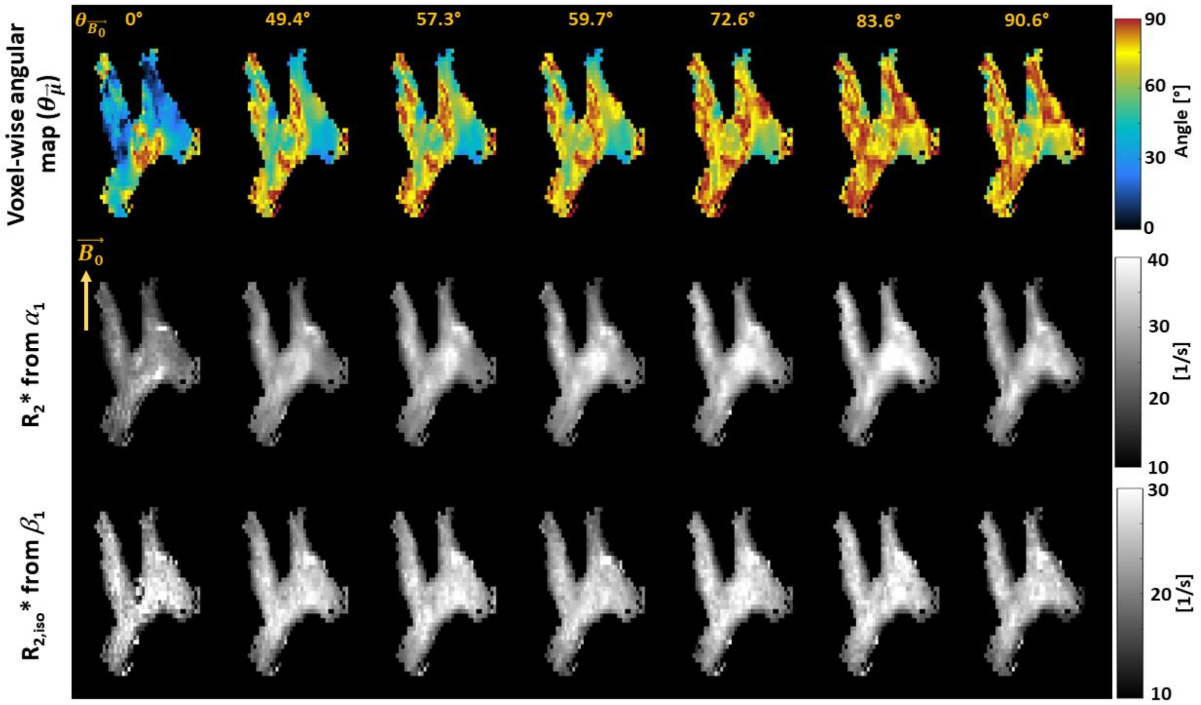

Figure S 1: R_2 angular ($\theta_{\vec{\mu}}$) dependency in a coronal section of the ex vivo specimen from 7 of the 16 angular measurements. They are sorted using the calculated $\overrightarrow{B_0}(\theta_i)$ angular elevation (θ_{B_0} in the inset) after coregistration: The first row shows the voxel-wise angular $\theta_{\vec{\mu}}$ map constrained between 0° and 90°. Second row is the estimated R_2* via α_1 parameter maps analysed with M1 (Equation 2). Third row shows the estimated $R_{2,iso}$* via β_1 parameter maps analysed with M2 (Equation 1).*

2. Hollow cylinder fibre model in detail

The hollow cylinder fibre model (HCFM) proposes an analytical approximation of the angular orientation ($\theta_{\vec{\mu}}$) dependence of the GRE signal to $\overrightarrow{B_0}$. This approximation establishes that the total MR signal comes from water molecules in an *infinitely long* hollow cylinder affected by the diamagnetic myelin sheath (Liu, 2010). Under this model, the diamagnetic myelin sheath magnetically is the only perturber of the water molecules and the myelin sheath divides the total volume in three distinguishable compartments: (1) the intra-axonal (S_A), (2) myelin (S_M) and (3) extra-cellular (S_E) compartments. Then, the total MR signal, S_C, is defined as:

$$S_C(t, \theta_{\vec{\mu}}) = S_A(t, \theta_{\vec{\mu}}) + S_E(t, \theta_{\vec{\mu}}) + S_M(t, \theta_{\vec{\mu}}), \qquad \text{(S1)}$$

where the signal decay coming from each compartment, as defined in (Wharton and Bowtell, 2012) and (Wharton and Bowtell, 2013), are defined as a function of time (t) and $\theta_{\vec{\mu}}$:

$$S_A(t,\theta_{\vec{\mu}}) \approx \rho_A V_A e^{-R_{2A}t+i\omega_A(\theta_{\vec{\mu}})t}, \quad \text{(S2a)}$$

$$S_E(t,\theta_{\vec{\mu}}) \approx \rho_E V_E e^{-R_{2E}t-D_E(t,\theta_{\vec{\mu}})}, \text{ and} \quad \text{(S2b)}$$

$$S_M(t,\theta_{\vec{\mu}}) \approx \rho_M V_M e^{-R_{2M}t+i\omega_M(\theta_{\vec{\mu}})t-D_M(t,\theta_{\vec{\mu}})}. \quad \text{(S2c)}$$

In these compartmental equations, ρ, R_2 and V are respectively the proton density, transverse relaxation rate and volumes for each compartment (defined with the corresponding sub-indices). The functions ω_A and ω_M are the (local) frequency offset of the intra-axonal and myelin water molecules produced by the myelin susceptibility (from (Wharton and Bowtell, 2012) and (Duyn, 2014)), defined as:

$$\omega_A(\theta_i) = \frac{-3\chi_A sin^2(\theta_i)}{4} ln(g_{ratio})\omega_0 \text{ and} \quad \text{(S3a)}$$

$$\omega_M(\theta_i) = \left(\frac{\chi_I}{2}\left(\frac{2}{3} - sin^2(\theta_i)\right) + \frac{\chi_A}{2}\left(\left(\frac{1}{4} + \frac{3g_{ratio}^2 ln(g_{ratio})}{2(1-g_{ratio}^2)}\right) sin^2(\theta_i) - \frac{1}{3}\right) + E\right)\omega_0, \quad \text{(S3b)}$$

where χ_I and χ_A are the isotropic and anisotropic magnetic susceptibilities of the myelin sheath (in ppm), E is the exchange factor between compartments (in ppm) and ω_0 is the Larmor frequency (= $\gamma|\overrightarrow{B_0}|$, in MHz, with γ the gyromagnetic ratio) of the water molecules. The D_E and D_M functions are the dephasing in the extracellular and myelin compartments across the voxel. D_E is defined, in the work of (Wharton and Bowtell, 2013), as a piece-wise function using the approximation introduced by (Yablonskiy and Haacke, 1994) and discussed in the following section. The D_M function is assumed to be negligible in this study, i.e., $D_M \approx 0$.

3. Analytical expression of the dephasing component (D_E) of the extracellular compartment (S_E)

Yablonskiy and Haacke, (1994) proposed the analytical expression for the magnetic dephasing of a medium due to the presence of cylindrical dephasors (defined as cylinders with a different magnetic susceptibility than the medium) oriented with an angle $\theta_{\vec{\mu}}$ to $\overrightarrow{B_0}$ defined as:

$$D_E(t,\theta_{\vec{\mu}}) = V_c \int_0^1 \left(\frac{1-J_0(\omega_E(\theta_{\vec{\mu}}, g_{ratio})tu)}{u^2}\right) du, \quad \text{(S4)}$$

where V_c is the cylinder's volume (equal to the fibre volume fraction, FVF), J_0 is the zeroth-order Bessel's function of the First Kind, u is the variable of integration and ω_E is the frequency offset in the extracellular space. The latter is defined as:

$$\omega_E(\theta_{\vec{\mu}}) = 2\pi\chi_E sin^2(\theta_{\vec{\mu}})\omega_0, \quad \text{(S5)}$$

where χ_E is the mean susceptibility of the myelin sheet, defined as $(\chi_I + 0.25\,\chi_A)(1 - g^2_{ratio})$. In the work of (Wharton and Bowtell, 2013; Yablonskiy and Haacke, 1994), Equation S4 was approximated for two-time scales divided by the so-called critical time (α in (Wharton and Bowtell, 2013)), defined as:

$$\alpha = 1.5 \cdot \omega_E^{-1}. \quad \text{(S6)}$$

For times shorter than the critical time, the dephasing function is approximated by a quadratic function, while for times longer than the critical time this function becomes linear. The corresponding analytical expressions (Yablonskiy and Haacke, 1994) are:

$$D_E(t,\theta_{\vec{\mu}}) = \begin{cases} \frac{FVF}{16}|\chi_E|^2 sin^4(\theta_{\vec{\mu}})\omega_0^2 t^2 = D_E' t^2, t < \alpha, \\ FVF\left(\frac{1}{2}|\chi_E| sin^2(\theta_{\vec{\mu}})t - 1\right) = D_E'' t - FVF, t > \alpha. \end{cases} \quad \text{(S7)}$$

In this equation, D_E' and D_E'' are expressions having all the parameters that are not time dependent, including $\sin^4(\theta_{\vec{\mu}})$ and $\sin^2(\theta_{\vec{\mu}})$, respectively. This simplified expression, especially the quadratic approximation, is used later (next section). However, this piecewise approximation has a discontinuity at this critical time, as observed in Figure S2. To avoid this discontinuity when D_E overpasses the critical time for the *in silico* data, we used an analytical solution to Equation S4. This solution was performed in Mathematica 12 (Wolfram Research, Inc., Champaign, IL (2020)), giving the following expression:

$$D_E(t,\theta_{\vec{\mu}}) = 0.5 \cdot FVF\left(-2 + \omega_E t J_1(\omega_E t)\left(-2 + \pi\omega_E t H_0(\omega_E t)\right) + J_0(\omega_E t)\left(2 + \left(2 - \pi H_1(\omega_E t)\right)(\omega_E t)^2\right)\right), \quad \text{(S8)}$$

in where J_1 is the first-order Bessel's function of the First-Kind, and H_0 and H_1 are the zeroth and first-order Struve functions ((Struve, 1882) and (Aarts and Janssen, 2016)), respectively. The offset frequency in the extracellular space (ω_E) is dependent on the mean angular orientation and the g-ratio, as defined in (Yablonskiy and Haacke, 1994) and (Wharton and Bowtell, 2012).

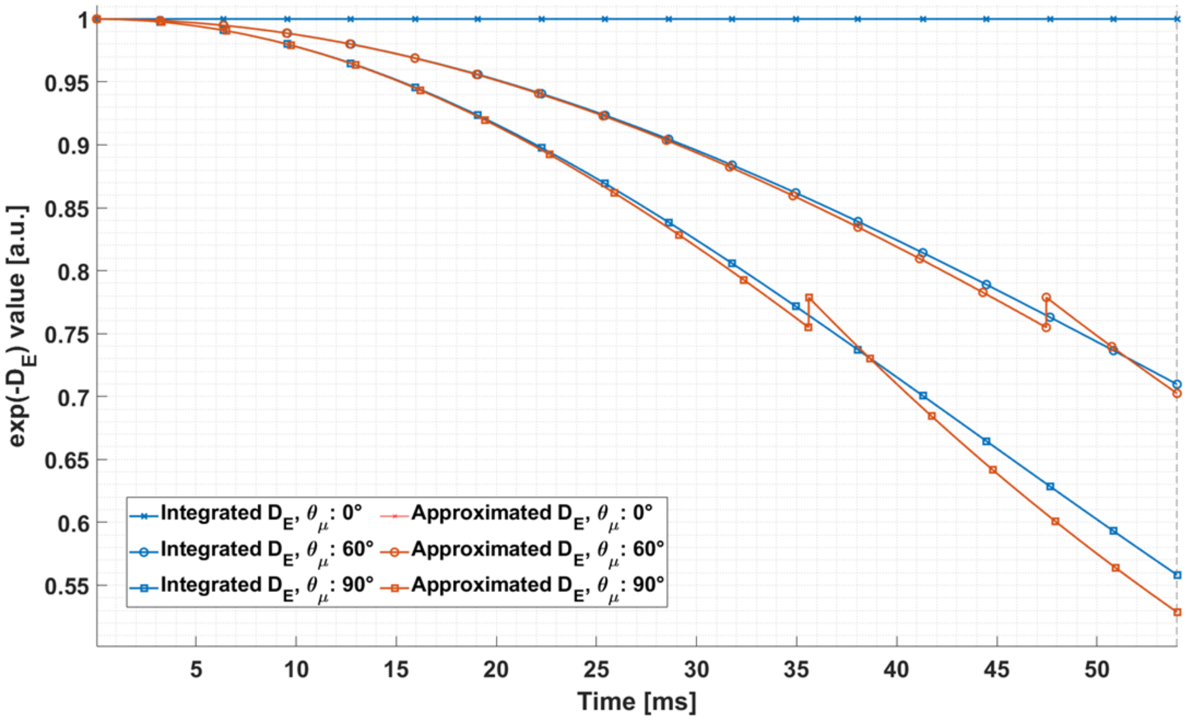

Figure S 2: Signal decay in the extra-cellular compartment due only to dephasing (D_E) using different D_E functions. The signal decay (i.e., exp(-D_E) in Equation S2b) was evaluated in function of time (in ms) and at three different angular orientations (0°, 60° and 90°). Two expressions for the D_E function (Equation S4) were used: the analytical solution given in Equation S8 (Integrated D_E, blue curve) and the piece-wise approximation proposed in the work of Yablonskiy et al. 1994 in Equation S7 (approximated D_E, orange curve). Both functions were evaluated using the simulation values (section 3.2, Table 1).

4. The magnitude of the signal decay in the hollow cylinder fibre model (HCFM) and it's second-order approximation, the log-quadratic model (M2)

The magnitude of the MR signal based on the HCFM is defined as:

$$|S_C| \approx \sqrt{R(S_C)^2 + I(S_C)^2}, \tag{S9}$$

where S_c is the total signal defined in Equation S1 and $R(.)$ and $I(.)$ are the real and imaginary components of S_c. Evaluating Equation S9 with Equations S1 and S2(a-c) results in:

$$|S_C| \approx \sqrt{\begin{matrix} V_A^2\rho_A^2 e^{-2R_{2A}t} + V_E^2\rho_E^2 e^{-2R_{2E}t-2D_E} + V_M^2\rho_M^2 e^{-2R_{2M}t-2D_M} \\ +2V_AV_E\rho_A\rho_E e^{-(R_{2A}+R_{2E})t-D_E}\cos(\omega_A t) \\ +2V_AV_M\rho_A\rho_M e^{-(R_{2A}+R_{2M})t-D_M}\cos\big((\omega_A-\omega_M)t\big) \\ +2V_EV_M\rho_E\rho_M e^{-(R_{2E}+R_{2M})t-D_E-D_M}\cos(\omega_M t) \end{matrix}}. \tag{S10}$$

Using the natural logarithm function (ln(x)) of the above equation and neglecting the contribution of D_M results in:

$$ln(|S_N|) \approx \frac{1}{2}log\begin{pmatrix} V_A^2\rho_A^2 e^{-2R_{2A}t} + V_E^2\rho_E^2 e^{-2R_{2E}t-2D_E} + V_M^2\rho_M^2 e^{-2R_{2M}t} \\ +2V_AV_E\rho_A\rho_E e^{-(R_{2A}+R_{2E})t-D_E}\cos(\omega_A t) \\ +2V_AV_M\rho_A\rho_M e^{-(R_{2A}+R_{2M})t}\cos\big((\omega_A-\omega_M)t\big) \\ +2V_EV_M\rho_E\rho_M e^{-(R_{2E}+R_{2M})t-D_E}\cos(\omega_M t) \end{pmatrix}. \tag{S11}$$

This expression can be approximated to a second-order polynomial model if the three functions related to time, i.e., the transverse relaxation rates (e.g. $e^{-R_2 t}$), the frequency offsets of the intra-axonal and myelin compartments (i.e. $\cos(\omega_A t)$ and $\cos(\omega_M t)$) and dephasing of the extra-axonal compartment (e.g. e^{-D_E}), are *sufficiently small*. Following the derivation of Wharton and Bowtell, 2013 (Wharton and Bowtell, 2013), the approximation of Equation S11 can be achieved if both exponential and cosine functions are expanded using the Taylor expansion in 2nd order:

$$e^{-x} \approx 1 - x + \frac{x^2}{2}, \text{ and} \tag{S12a}$$

$$cos(x) \approx 1 - \frac{x^2}{2}. \tag{S12b}$$

If these conditions are fulfilled and the quadratic approximation for D_E is used ($D_E = D_E' t^2$, Equation S7), the argument of the logarithm function in Equation S11 *up to order two in time* becomes:

$$ln(|S_N|) \approx \tag{S13}$$

$$\frac{1}{2}log\begin{pmatrix} (V_A\rho_A + V_E\rho_E + V_M\rho_M)^2 - 2(V_A\rho_A + V_E\rho_E + V_M\rho_M)(V_A\rho_A R_{2A} + V_M\rho_M R_{2M} + V_E\rho_E R_{2E})t \\ -[2(V_A\rho_A + V_E\rho_E + V_M\rho_M)V_E\rho_E D_E' - 2V_A^2\rho_A^2R_{2A}^2 - 2V_M^2\rho_M^2R_{2M}^2 - 2V_E^2\rho_E^2R_{2E}^2 \\ +2V_AV_E\rho_A\rho_E\left(\frac{(\omega_A)^2}{2} - \frac{[(R_{2A}+R_{2E})]^2}{2}\right) \\ +2V_AV_M\rho_A\rho_M\left(\frac{((\omega_A-\omega_M))^2}{2} - \frac{[(R_{2A}+R_{2M})]^2}{2}\right) \\ +2V_EV_M\rho_E\rho_M\left(\frac{(\omega_M)^2}{2} - \frac{[(R_{2M}+R_{2E})]^2}{2}\right)]t^2 + \mathcal{O}(t^3) \end{pmatrix}.$$

The expression of Equation S13 can be approximated by a 2nd order Taylor expansion in time[1]:

$$ln(|S_N|) \approx \gamma_0 - \gamma_1 t - \gamma_2 t^2, \quad \text{(S14)}$$

in which each γ-parameter is defined as:

$$\gamma_0 = \ln(V_A\rho_A + V_E\rho_E + V_M\rho_M) = \ln(\langle V\rangle_\rho), \quad \text{(S15a)}$$

$$\gamma_1 = \frac{V_A\rho_A R_{2A} + V_E\rho_E R_{2E} + V_M\rho_M R_{2M}}{V_A\rho_A + V_E\rho_E + V_M\rho_M} = \frac{\langle R_2\rangle_{\rho,V}}{\langle V\rangle_\rho}, \quad \text{(S15b)}$$

$$\gamma_2 = \frac{2\nu_E D_E'\langle V\rangle_\rho - \nu_A\left(\nu_E(\Delta R_{2AE}^2 - \omega_A^2) + \nu_M(\Delta R_{2AM}^2 - \omega_{AM}^2) - \nu_M\nu_E(\Delta R_{2EM}^2 - \omega_M^2)\right)}{2\langle V\rangle_\rho^2}; \quad \text{(S15c)}$$

and, for simplification, $\Delta R_{2ij} = R_{2i} - R_{2j}$, $\Delta\omega_{ij} = \omega_i - \omega_j$ and $\nu_i = V_i\rho_i$ (sub-indexes i and j are the compartment's indices A, E and M). Note that, in contrast to (Wharton and Bowtell, 2013), we did not neglect the contribution of the myelin-water signal at this point. Thus, the expression in Equation S14 can describe the magnitude of the signal decay of the HCFM by including all the compartments on under certain conditions: (1) the signal of the myelin compartment is completely diminished for echo times longer than T_{2M}, therefore its contribution must be discarded after the corresponding echo time (i.e., $\nu_M \approx 0$ for TE = T_{2M}). This also ensures that the approximation of Equation S12a remains valid for TE larger than T_{2M}. And (2), given the parameters used in this simulation (section 3.2, Table 1), the accuracy of Equation S14 decreases at TE > 30 ms (not shown here).

With this linear approximation of the HCFM, it is clear that the log-quadratic model (M2) can be defined as a simplification of Equation S14 and the M2 parameters of Equations S15a-d by neglecting the contribution of myelin (i.e. $\nu_M \approx 0$). If so, the parameters of Equation S14 can be simplified in the following equation:

$$M2: ln(|S_N|) \approx \beta_0 - \beta_1 t - \beta_2 t^2, \quad \text{(S16)}$$

Where:

$$\beta_0 = log(V_A\rho_A + V_E\rho_E), \quad \text{(S17a)}$$

$$\beta_1 = \frac{V_E\rho_E R_{2E} + V_A\rho_A R_{2A}}{(V_A\rho_A + V_E\rho_E)}, \quad \text{(S17b)}$$

$$\beta_2 = \frac{\nu_E(\nu_A(\omega_A^2 + 2D_E' - (\Delta R_{2AE})^2) + 2\nu_E D_E')}{2(\nu_A + \nu_E)^2}. \quad \text{(S17c)}$$

In the scenario where R_{2A} is equal to R_{2E} (i.e. $R_{2A} = R_{2E} \equiv R_{2N}$, with N denoting "non-myelinated"), the analytical expression for β_1 becomes $\beta_{1,nm}$ (Equation 3, section 2.2).

The proposed heuristic analytical expression of β_1 in Equation 4, $\beta_{1,m}$, was motivated originally by taking Equation S17b and incorporating the myelin compartment information (V_M, ρ_M and R_{2M}) in a similar manner, resulting in the following expression:

$$\beta_1 = \frac{V_E\rho_E R_{2E} + V_A\rho_A R_{2A} + V_M\rho_M R_{2M}}{(V_A\rho_A + V_E\rho_E + V_M\rho_M)}, \quad \text{(S18)}$$

---

[1] Note that the transition from Equation S13 to S14 was possible by performing a Taylor's expansion of the function $\log(a + bt + ct^2)$. This expansion results in the expression $\log(a) + \frac{b}{a}t + \frac{b^2 - 2ac}{2a^2}t^2$, which are the corresponding parameters defined in Equations S15a-c.

which is the same expression derived in Equation S15b. Here we propose β_1 in Equation S18 (or $\beta_{1,m}$ in Equation 6 under the assumptions: $R_{2A} = R_{2E} \equiv R_{2N}$, $\rho_A = \rho_E \equiv \rho_N$, and $V_A + V_E = 1 - V_M$) as a heuristic term rather a derived term from the HCFM (i.e. γ_1, Equation S15b) given the validity range of Equation S14. Surprisingly, it turned out that this heuristic parameter β_1 explains better the *in silico* fitted β_1 than the $\beta_{1,nm}$ (Equations 3 and S17b, see Figure 7).

Equation S18 can be re-written as a function of the myelin water fraction (MWF), axonal water fraction (AWF) and extra-axonal water fraction (EWF), defined as:

$$MWF = \frac{\rho_M v_M}{\rho_A v_A + \rho_E v_E + \rho_M v_M}, \tag{S19a}$$

$$AWF = \frac{\rho_A v_A}{\rho_A v_A + \rho_E v_E + \rho_M v_M}, \tag{S19b}$$

$$EWF = \frac{\rho_E v_E}{\rho_A v_A + \rho_E v_E + \rho_M v_M}. \tag{S19c}$$

Since the sum of the water fractions are equal to 1, β_1 becomes:

$$\beta_1 = MWF \cdot R_{2M} + AWF \cdot R_{2A} + EWF \cdot R_{2E}, \text{ or} \tag{S20a}$$

$$\beta_1 = MWF \cdot R_{2M} + (1 - MWF) \cdot R_{2N}; \tag{S20b}$$

where Equation 5 (or S20b) is obtained if we assume in Equation S20a that the relaxation rate in the intra- and extra-cellular water is the same: $R_{2A} = R_{2E} \equiv R_{2N}$.

5. *In silico* data setup: SNR and irregular binning

To make the *in silico* data as similar as possible to the *ex vivo* data, noise was added to the signal decay of the *in silico* data, in such a way that the *in silico* SNR is like the SNR seen in the *ex vivo* GRE data. For that, the *ex vivo* SNR was calculated by dividing the signal of the white matter region of the OC and the standard deviation of the background in (image) magnitude space. No noise correlation correction was performed in this calculation since the coil has only 2 receiver channels. As a result, an average SNR value of 112 was obtained for this region (section 3.2), and with this SNR value, a complex random Gaussian noise was added to the *in silico* data as follows:

$$S_{silico}(t, SNR = 112) = S_{silico}(t, SNR = \infty) + N\big(0, \sigma_{silico}(SNR = 112)\big) + \big(0, \sigma_{silico}(SNR = 112)\big), \tag{S21}$$

where N(0,σ) is the Normal distribution with mean 0 and the standard deviation defined by:

$$\sigma_{silico}(SNR) = \frac{|S_{silico}(t=0)|}{SNR}, \tag{S22}$$

where the magnitude signal is divided by the desired SNR at time 0 ($|S_{silico}(t = 0)|$).

With the noise added, the magnitude of the *in silico* MR signal at SNR = 112 was obtained:

$$|S_{Silico}| = \sqrt{R(S_{silico})^2 + I(S_{silico})^2}. \tag{S23}$$

To compare the *in silico* data analysis across the 5000 signal decays per simulated g-ratio, sampled κ and $\theta_{\vec{\mu}}$ to the irregularly binned *ex vivo* data analysis (section 3.3.1 and Figure 5B), the α-parameters and β-parameters from the *in silico* data required three consecutive averaging-steps: (1)

an averaging across the 5000 replicas, resulting in the replica-averaged $\widehat{\alpha_l}(i: 0,1)$, $\widehat{\beta_J}(j: 0,1,2)$ and their standard deviations $sd(\alpha_i), sd(\beta_j)$ per sampled κ value and $\theta_{\vec{\mu}}$. (2) A weighted averaging across κ values per each $\theta_{\vec{\mu}}$ irregular bin of the *ex vivo* data in each κ range. For that, it was obtained the distribution of the κ values from the voxels contained in each of the 20 defined $\theta_{\vec{\mu}}$ irregular bins. The $\theta_{\vec{\mu}}$ range per bin and κ range is given in Table A1. Then, all the obtained distributions were averaged per κ range (Figure S3 from A to C) to remove possible influence of the irregular $\theta_{\vec{\mu}}$ bins on κ. The standard deviation from this average was calculated, normalised and used later (referred as the sd($P(\kappa_l)$) in Equation S28). Next, a probability distribution, $P(\kappa_l)$, was fitted accordingly (Figure S3 from D to F) and the weighted averaging on $\widehat{\beta_J}$ (the same procedure is performed for $\widehat{\alpha_l}$) was calculated as follows:

$$\langle\beta_j\rangle_P = \frac{\sum_l \widehat{\beta_J}(\kappa_l)P(\kappa_l)}{\sum_l P(\kappa_l)}, \tag{S24}$$

where the expression for $P(\kappa_l)$ was heuristically chosen and varied per each fibre dispersion (κ range): a Beta distribution for the highly dispersed fibres (κ < 1 range, Figure S3-D), defined as:

$$P(\kappa_l < 1) = \frac{\kappa_l^{a-1}(1-\kappa_l)^{b-1}\Gamma(a+b)}{\Gamma(a)\Gamma(b)}, \tag{S25}$$

$$\text{where } \Gamma(c) = \int_0^\infty x^{c-1}e^{-x}dx \tag{S26}$$

is the Gamma function. The coefficients a and b estimated for this range were 3.145 and 1.234, respectively. Given the clear half-shaped normal distribution, a Half-Normal distribution for the mildly dispersed fibres (1 ≤ κ < 2.5 range, Figure S3-E) was used, defined as:

$$P(1 \le \kappa_l < 2.5) = \frac{\sqrt{2}}{\sigma\sqrt{\pi}} exp\left(\frac{-(\kappa_l-1)^2}{2\sigma^2}\right). \tag{S27}$$

The coefficients μ and σ were 0 and 0.4498, respectively. And given the fast decay of the values at the beginning of the distribution, an Exponential distribution for the highly aligned fibres (2.5 ≤ κ range, Figure S3-F) was used, defined as:

$$P(2.5 \le \kappa_l) = \lambda exp\big(-\lambda(\kappa_l - 2.5)\big). \tag{S28}$$

The coefficient λ was 0.2241. The standard deviation of $\langle\beta_j\rangle_P$ was also estimated by error-propagating the $sd(\beta_j)$ weighted by $P(\kappa_l)$ and its standard deviation sd($P(\kappa_l)$), as follows:

$$sd\left(\langle\beta_j\rangle_P\right) = \sqrt{\sum_l \left(\frac{sd\big(\beta_j(\kappa_l)\big)P(\kappa_l)}{\sum_l P(\kappa_l)}\right)^2 + \left(\widehat{\beta_J}(\kappa_l)\, sd\big(P(\kappa_l)\big)\right)^2}, \tag{S29}$$

while the first squared term requires the normalisation factor ($\sum_l P(\kappa_l)$) because the weights $P(\kappa_l)$ are not normalised, the second is not needed since sd($P(\kappa_l)$) is already normalised. Finally, the $\langle\beta_j\rangle_P$ and $sd\left(\langle\beta_j\rangle_P\right)$, and the $\langle\alpha_i\rangle_P$ and $sd(\langle\alpha_i\rangle_P)$ (as in Equation S29) were averaged and error-propagated, respectively, as a function of the $\theta_{\vec{\mu}}$ values for each irregular bin.

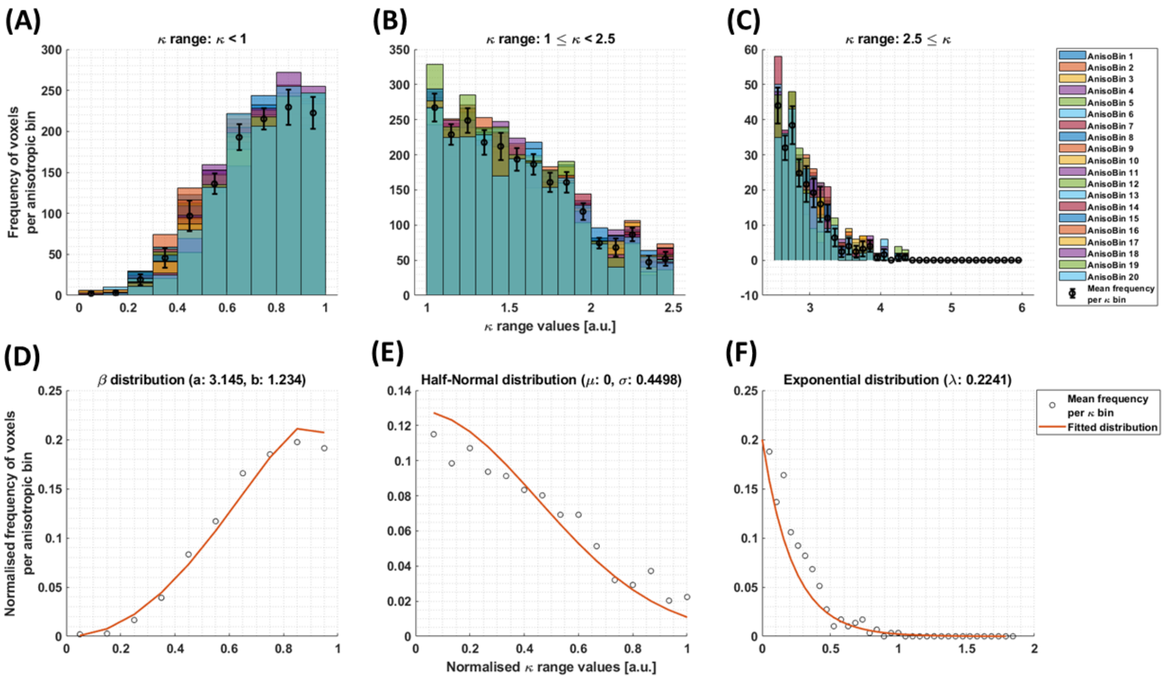

Figure S3: Assembling the in silico data across the simulated κ ranges and angular ($\theta_{\vec{\mu}}$) irregular bins. To make the in silico data comparable to the ex vivo data, the frequency of voxels as a function of κ was obtained per defined $\theta_{\vec{\mu}}$ irregular bin in the ex vivo data (Figure 3). This was performed for the 20 $\theta_{\vec{\mu}}$ bins (AnisoBin X, with X the corresponding bin from 1 to 20, see Table A1) and per fibre dispersion (κ range): highly dispersed (κ < 1, A), mildly dispersed (1 ≤ κ < 2.5, B) and negligibly dispersed (κ ≥ 2.5, C) fibres. The mean and standard deviation across histograms were obtained (error bars). The means were normalised with respect to the cumulated value (i.e., sum of all the mean values) and fitted with a continuous function ($P(\kappa_l)$) per κ range, previously normalised: a beta distribution for κ < 1 (D), half-normal distribution for 1 ≤ κ < 2.5 (E) and exponential distribution for κ ≥ 2.5 (F). The standard deviation was also normalised by the cumulated value per κ range and used as the standard deviation of the continuous distributions (sd($P(\kappa_l)$)).

Dispersion range / Irregular bin	1 < κ	1 ≤ κ < 2.5	2.5 ≤ κ
#1 and (θ_0)	[0, 29.3]° (17.3°)	[0, 26.5]° (20.4°)	[0, 30.7]° (22.9°)
#2	[29.3, 39.3]°	[26.6, 36.8]°	[30.8, 40.8]°
#3	[39.4, 45.9]°	[36.9, 43.5]°	[40.9, 47.2]°
#4	[45.6, 50.8]°	[43.6, 48.6]°	[47.3, 52.1]°
#5	[50.9, 55.0]°	[48.7, 52.9]°	[52.2, 56.2]°
#6	[55.1, 58.6]°	[53.0, 56.6]°	[56.3, 59.7]°
#7	[58.7, 61.8]°	[56.7, 60.0]°	[59.8, 62.7]°
#8	[61.9, 64.7]°	[60.1, 63.0]°	[62.8, 65.6]°
#9	[64.8, 67.4]°	[63.1, 65.9]°	[65.7, 68.3]°
#10	[67.5, 70.0]°	[66.0, 68.5]°	[68.4, 70.7]°
#11	[70.1, 72.3]°	[68.6, 71.0]°	[70.8, 72.9]°
#12	[72.4, 74.6]°	[71.1, 73.5]°	[73.0, 75.2]°
#13	[74.7, 76.7]°	[73.6, 75.7]°	[75.3, 77.2]°
#14	[76.8, 78.7]°	[75.6, 77.8]°	[77.3, 79.2]°
#15	[78.8, 80.6]°	[77.9, 79.8]°	[79.3, 81.1]°
#16	[80.7, 82.4]°	[79.9, 81.7]°	[81.2, 82.8]°
#17	[82.5, 84.1]°	[81.8, 83.6]°	[82.9, 84.4]°
#18	[84.2, 85.7]°	[83.7, 85.3]°	[84.5, 85.9]°
#19	[85.8, 87.3]°	[85.4, 87.3]°	[86.0, 87.4]°

#20	[87.4, 90]°	[87.4, 90]°	[87.5, 90]

Table S1 : Range of angles ($\theta_{\vec{\mu}}$) defined by [min, max] values, contained in each $\theta_{\vec{\mu}}$ irregular bin per fibre dispersion (κ range) in the ex vivo data (section 3.3.1). The angular offset, θ_0 (see section 3.3.1), is defined as the angular average of the 1st irregular bin, resulting in 17.3° (κ < 1), 20.4° (1 ≤ κ < 2.5) and 22.9° (2.5 ≤ κ).

6. The weighted-Akaike Information Criterion for the studied models

To assess whether the increased model complexity of M2 is better explained than M1 by the meGRE signal decay, the weighted-Akaike Information Criterion corrected was calculated (wAICc, (Burnham et al., 2011)). The wAICc was estimated based on the difference of the corrected Akaike Information Criterion (AICc) between the models under study. In general, the wAICc for a given set of j models is defined as:

$$\mathrm{wAICc}(\mathrm{M_l}) = \frac{\exp\left(-0.5\,\Delta\mathrm{AICc}(\mathrm{M_l})\right)}{\sum_j \exp\left(-0.5\,\Delta\mathrm{AICc}(\mathrm{M_j})\right)}, \tag{S30}$$

where ΔAICc is the difference of two AICcs defined as:

$$\Delta\mathrm{AICc}(\mathrm{M_l}) = \mathrm{AICc}(\mathrm{M_l}) - \min_{M_j \in \{M_j\}_j} (\mathrm{AICc}(\mathrm{M_j})), \tag{S31}$$

and the corresponding AICc per model is defined as:

$$\mathrm{AICc}(\mathrm{M_l}) = n\,log\left(\frac{\mathrm{SSE}(\mathrm{M_l})}{n}\right) + 2k + \frac{2k(k+1)}{n-k-1}, \tag{S32}$$

where SSE is the sum of squared errors between the data points and the fitted model $\mathrm{M_l}$, n is the sample size and k is the number of parameters in each model, when the data is being fitted by linear regression (Gordon, 2010).

In the following, we consider two models: M_1 = M1 (Equation 2 with k = 2) and M_2 = M2 (Equation 1 with k = 3). The work of (Burnham et al., 2011) classifies how a model is being described by the data based on the ΔAICc. In our conservative approach (section 3.3.2), we reduced the original classification of ΔAICc based on how M2 could be explained by the data and take the so-called *ΔAICc > 2 Rule* to determine the threshold. First, we explored the case where AICc(M1) > AICc(M2). In this case, the wAICc for M2 is defined as (according to Equation S30):

$$\mathrm{wAICc}(\mathrm{M_2}) = \frac{1}{(1+\exp(-0.5\Delta\mathrm{AIC(M1)}))}, \text{ with} \tag{S33}$$

$$\Delta\mathrm{AICc(M1)} = \mathrm{AICc(M1)} - \mathrm{AICc(M2)} \text{ and } \Delta\mathrm{AICc(M2)} = 0 \tag{S34}$$

which are the Equations 11 and 12 used in section 3.3.2. Note that for the case AICc(M1) ≤ AICc(M2), we considered that M2 is not being explained by the data as compared to M1. Second, the *ΔAICc > 2 Rule* is what early studies used to dismiss a model of a study. The corresponding wAICc value for the dismissed model is 0.27, while the wAICc value for the best model is 0.73. It is important to mention that (Burnham et al., 2011) discussed that this cutoff is really poor. However, we decided to use this conservative rule for our study.

Here, we use the wAICc to test how well the data are explained by a given model as compared to another. It is important to mention, according to (Burnham et al., 2011), that a high wAICc for a specific model does not mean that this particular model fits the data the best, instead it suggests that this model is favourable (as compared to another model) by the data in the trade-off between bias and variance of the fitted model parameters given a specific dataset and sample size.

7. R_2* toolbox and *in silico* dataset using Wharton and Bowtell's *in vivo* compartmental R_2s values

A self-made MATLAB code capable of simulating and analyzing multiecho gradient echo (meGRE) MR signal based on a user-defined set of parameters is presented in this work. The code provides a versatile tool to gain insights into the underlying processes associated with meGRE MR data. By allowing users to define input parameters like the ones defined in Table 1 in the main manuscript, the simulation can be tailored to specific research needs, facilitating a comprehensive analysis of R_2* behavior under various scenarios. This code gives as output all the analyses presented in the main manuscript, particularly the first, second and fourth analyses. This self-developed MATLAB code empowers researchers to deepen their understanding and make informed decisions based on the simulated and analysed R_2* using the classical M1 or log-quadratic M2 models. This code is free and available to use in the following Github repository: https://github.com/quantitative-mri-and-in-vivo-histology/r2s_iso_estimation

To demonstrate the capabilities of the MATLAB code, new *in silico* data was generated using the R_2 values from (Wharton and Bowtell, 2012). These results complement the found results for the *in silico* dataset simulated with R_2 values from (Dula et al., 2010).

7.1. Methods: in silico dataset

7.1.1. Simulated R_2* signal decay from the HCFM

Multi-echo GRE signal decay was simulated as ground truth (hereafter, *in silico* data) to assess the impact on M2 of variable fibre orientation, dispersion and myelination (i.e., g-ratio). The averaged MR signal, assumptions, addition of dispersion and noise were performed equally as in section 3.2. The main difference is the R_2 values per compartment, obtained from (Wharton and Bowtell, 2012): R_{2N} of 27.8 s^{-1} and R_{2M} of 125 s^{-1}.

7.1.2. Data analysis

The generated *in silico* data was fitted and binned equally as in section 3.3.1. The quantitative analyses were also performed as described in section 3.3.2 and their corresponding subsections.

7.2. Results

7.2.1. First analysis: Ability of M2 to obtain the angular-independent β_1 parameter for varying g-ratio and fibre dispersion values

Figure S4 shows the performance of M2 when estimating $R_{2,iso}$* via β_1 for variable g-ratio and fibre dispersion. To visualise this, we compared the $\theta_{\vec{\mu}}$ dependence of α_1 from M1 to the residual $\theta_{\vec{\mu}}$ dependence of β_1 from M2 (Figure S4A and S4B). Both $\theta_{\vec{\mu}}$ dependencies were quantified in Figure S4C using their respective nRMSD (Equation 9). The results are from the analysis performed on the *in silico* and *ex vivo* data.

The ability of M2 to reduce the $\theta_{\vec{\mu}}$ dependency of β_1 varied with g-ratio and fibre dispersion, the $\theta_{\vec{\mu}}$ dependency of α_1 was also strongly influenced by g-ratio and fibre dispersion: smaller g-ratio values and reduced fibre dispersion increased the $\theta_{\vec{\mu}}$ dependency of α_1 and (the residual $\theta_{\vec{\mu}}$ dependency) of β_1 (Figure S4A and S4B, respectively).

The fibre dispersion affected the performance of M2 similarly between *in silico* and *ex vivo* datasets (Figure S4C bottom). In both datasets, the improvement is largest for negligible

dispersion (from ΔnRMSD = -13.1%-points for the *in silico* data with a g-ratio of 0.8 and ΔnRMSD = -37.6%-points for the *ex vivo* data). For the *ex vivo* data, the nRMSD(β_1) was the lowest for the negligibly dispersed fibres (nRMSD(β_1): 1.3% at κ ≥ 2.5). For the *in silico* data, the nRMSD(β_1) was the lowest for the highly dispersed fibres and for a g-ratio of 0.8 (nRMSD(β_1): 0.11% to 0.21% with decreasing fibre dispersion). For the g-ratios of 0.66 and 0.73, the nRMSD(β_1) was higher but still below 19%.

The $\theta_{\vec{\mu}}$ dependence of α_1 on fibre dispersion was the same between *in silico* and *ex vivo* datasets (Figure S4C top): the lower the dispersion the higher the nRMSD(α_1). The $\theta_{\vec{\mu}}$ dependence of α_1 increased as the g-ratio decreased.

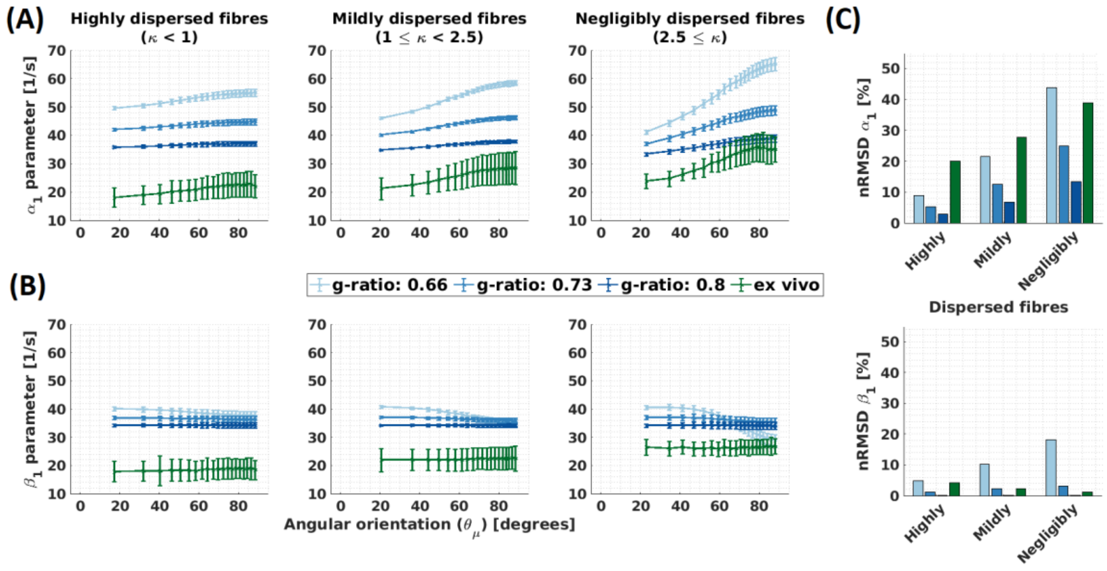

Figure S 4: Orientation dependence and quantification of linear model parameters (α_1 and β_1) for varying g-ratio and fibre dispersion values. (A-B) Depicted is the α_1 parameter of M1 (proxy for R_2) and β_1 parameter of M2 (proxy for the isotropic part of R_2*) as a function of the angle between the main magnetic field and the fibre orientation ($\theta_{\vec{\mu}}$) for different fibre dispersion and g-ratio values. The different columns depict different dispersion regimes: highly dispersed (κ < 1, first column), mildly dispersed (1 ≤ κ < 2.5, second column) and negligibly dispersed (κ ≥ 2.5, third column) fibres. Note that the smallest angle (θ_0) varied across dispersion regimes: 17.3° (κ < 1), 20.4° (1 ≤ κ < 2.5) and 22.9° (2.5 ≤ κ). This was caused by the irregular binning (see section 3.1.4). (C) Depicted is the normalised root-mean-squared deviation (nRMSD, Equation 11 in %) of the α_1 parameter of M1 (proxy for R_2*, top) and β_1 parameter of M2 (proxy for the isotropic part of R_2*, bottom) for different fibre dispersion and g-ratio values. The distinct colours of the curves in A-B or bars in C (blue and green) distinguish between in silico data with variable g-ratios (increasing blue hue with increasing g-ratio) and ex vivo data (olive curve).*

7.2.2. Second analysis: Assessment of the microstructural interpretability of β_1

Figure S5A and S5B report the angular-orientation ($\theta_{\vec{\mu}}$) dependent relative differences (ϵ_{nm} and ϵ_m, Equation 11) between the fitted β_1 from the *in silico* data and its predicted counterparts using M2 (Equation 3) and the heuristic expression (Equation 4). Figure S5C shows the mean and standard deviation of ϵ_{nm} and ϵ_m across angles.

ϵ_{nm} was large, between -50% and 0%, and varied strongly with g-ratio and fibre dispersion. Even more, ϵ_{nm} showed a $\theta_{\vec{\mu}}$ dependence where the largest deviation was observed for the smallest g-ratio (0.66) and the lowest fibre dispersion (Figure S5A). By contrast, ϵ_m was smaller across all the studied fibre dispersions, between 10% and 41%, and showed a smaller $\theta_{\vec{\mu}}$ dependence, which was largest for the smallest g-ratio and lowest fibre dispersion. However, on average, we found that negligibly dispersed fibres showed the smallest ϵ_{nm} and ϵ_m per g-ratio.

The mean across angles for ϵ_{nm}, $\langle\epsilon_{nm}\rangle$, was higher than -20% whereas the mean across angles for ϵ_m, $\langle\epsilon_m\rangle$, was higher than 15% (Figure S5C). On average across all g-ratios and fibre dispersion arrangements, $\langle\epsilon_{nm}\rangle$ was approximately 2 times larger than $\langle\epsilon_m\rangle$, except for the negligibly dispersed fibres, in where $\langle\epsilon_m\rangle$ was approximately 1.5 larger than $\langle\epsilon_{nm}\rangle$. Interestingly, the highest changes in the relative differences was observed at g-ratio of 0.66, in which $\langle\epsilon_{nm}\rangle$ decreased and $\langle\epsilon_m\rangle$ increased with decreasing fibre dispersion. Simultaneously, the variability for both relative mean differences, $sd(\epsilon_{nm})$ and $sd(\epsilon_m)$ respectively, increased; indicating the strong residual $\theta_{\vec{\mu}}$-dependency on the β_1 parameters.

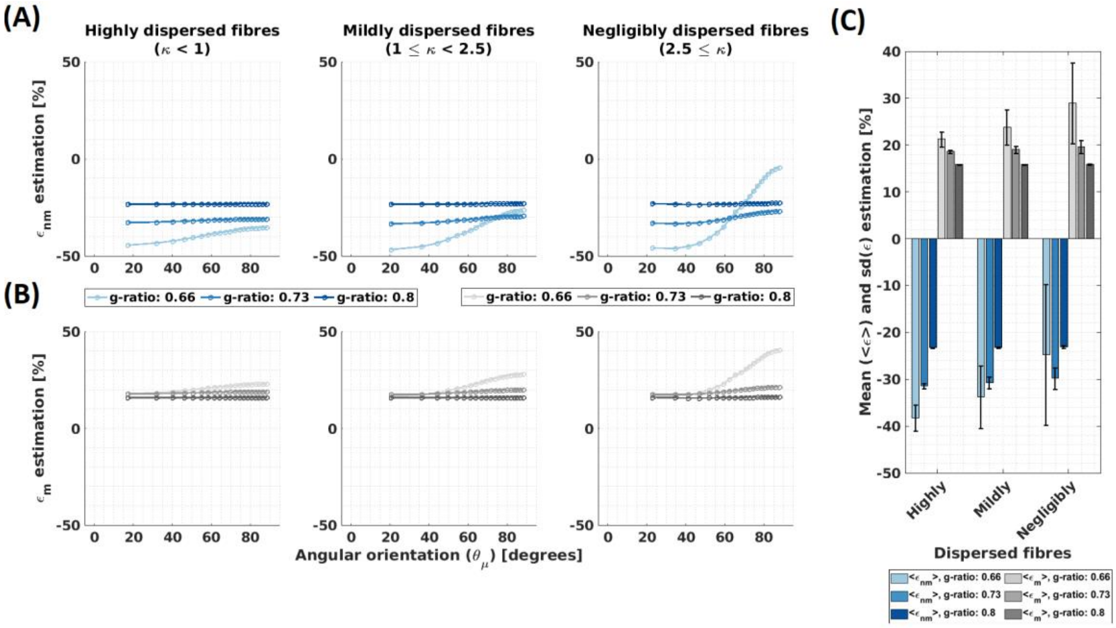

Figure S 5: Assessment of the microstructural interpretability of β_1 by the deviation between fitted and biophysically predicted β_1. The relative difference (ε, Equation 11) was calculated between the fitted β_1 to the in silico data and two biophysically-modelled expressions for β_1 based on the HCFM. The two expressions for β_1 values were calculated from the original expression for M2, $\beta_{1,\mathrm{nm}}$ (Equation 3, resulting in ε_{nm}) and the heuristic expression, $\beta_{1,\mathrm{m}}$ (Equation 4, resulting in ε_m). This was calculated per g-ratio and fibre dispersion. (C) The corresponding mean, <ε>, and standard deviation, sd(ε), of the relative differences across the angular orientations ($\theta_{\vec{\mu}}$) were estimated. The hue intensity coding represents increasing g-ratio value for both error estimations.

7.2.3. Third analysis: Myelin water fraction (MWF) and g-ratio estimation from ex vivo data using the heuristic expression of $R_{2,iso}$* via $\beta_{1,m}$

Figure S6 reports the MWF estimated from the *ex vivo* data by inverting the heuristic expression for $\beta_{1,\mathrm{m}}$ (Equation 6), using the compartmental R_2 values from (Wharton and Bowtell, 2012) (Table 1). Figure S6A shows the estimated MWF as a function of $\theta_{\vec{\mu}}$ while Figure S6B shows the median and standard deviation (sd) of the estimated MWF across $\theta_{\vec{\mu}}$.

The estimated MWF was larger with decreasing fibre dispersion (Figures S6A). Moreover, there was a trend towards larger estimated MWF for larger $\theta_{\vec{\mu}}$. Across $\theta_{\vec{\mu}}$, the estimated median *ex vivo* MWF was -0.01 for fibres with negligible dispersion but moved towards to even lower small values (MWF: -0.09) for dispersed fibres (Figure S6B, respectively). The standard deviation across MWF was similar for different fibre dispersions, ranging from 0.0068 to 0.0104.

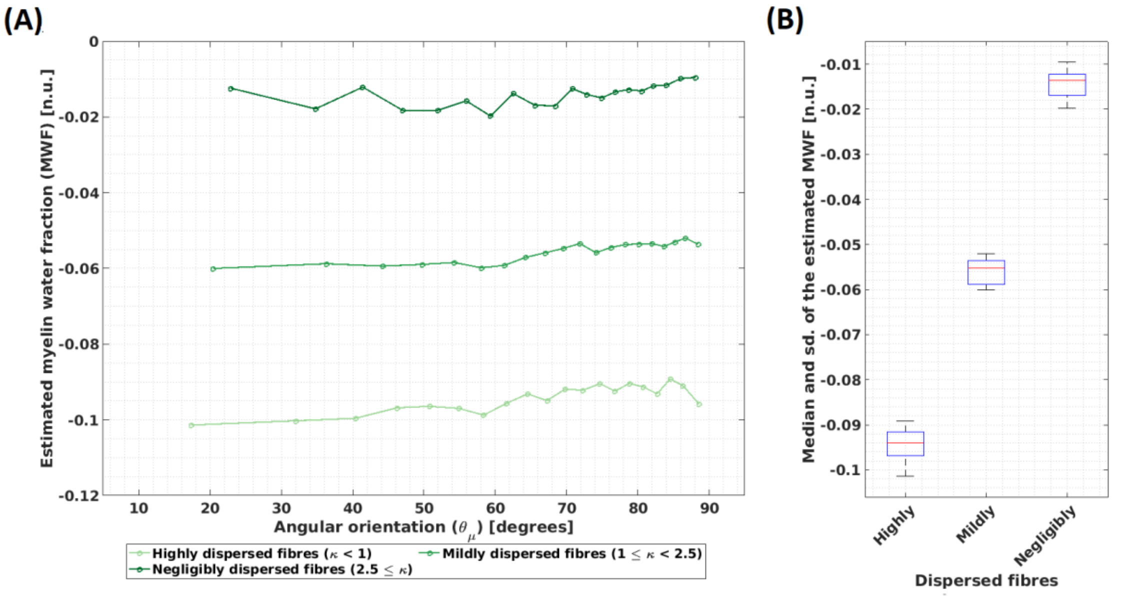

Figure S6: Dependence of the MWF estimation on angular orientation for three different fibre dispersion ranges in ex vivo data. (A) The MWF was estimated by using the heuristic analytical expression of β1 ($\beta_{1,m}$, Equation 4) and the fitted β_1 for the ex vivo data using the compartmental R_2 values from Wharton and Bowtell, 2013 (hues of green) in Table 1. This calculation was performed per angle ($\theta_{\vec{\mu}}$) and for the three different fibre dispersion ranges: highly dispersed, mildly dispersed and negligibly dispersed. The increasing green hue represents decreasing fibre dispersion. (B) The corresponding median and standard deviation (sd) were estimated across $\theta_{\vec{\mu}}$ per fibre dispersion range.

7.2.4. Fourth analysis: the effect of echo time ranges on the performance of M2

In this section, two sub-analyses were performed for *in silico* data at variable g-ratio and *ex vivo* data, both with negligibly dispersed fibres (i.e., κ ≥ 2.5) using the three meGRE subsets with different maximum echo time (TE_{max}). In the first sub-analysis, we presented similar results as in Figure 6, only for different TE_{max}. In the second sub-analysis, it was assessed whether M2 was better explained than M1 by the different meGRE subsets using the corresponding average wAICc of M2 (Equation 12).

7.2.4.1. First sub-analysis: assessing the residual $\theta_{\vec{\mu}}$ dependence in β_1 subsets with different maximum echo times

Using the meGRE subset with the largest TE_{max}, the $\theta_{\vec{\mu}}$ dependence of α_1 and residual $\theta_{\vec{\mu}}$ dependence of β_1 were similar for *ex vivo* and *in silico* data (Figure S7, first column). M2 could greatly reduce the $\theta_{\vec{\mu}}$ dependency of β_1 when compared to the $\theta_{\vec{\mu}}$ dependency of α_1 (Figure S7C): nRMSD(α_1) of 15.7% (*in silico*) and 37.9% (*ex vivo*) was reduced to 3.8% (*in silico*) and 1.3% (*ex vivo*). Using the meGRE subsets with smaller TE_{max} (36 ms and smaller), M2 was less effective (Figure S7A-B, second and third column). An increased $\theta_{\vec{\mu}}$ dependence was observed for β_1 compared to α_1 with an ΔnRMSD = 5.3%-points at 36 ms (*in silico*) and ΔnRMSD = 14.1%-points at 18 ms (*ex vivo*).

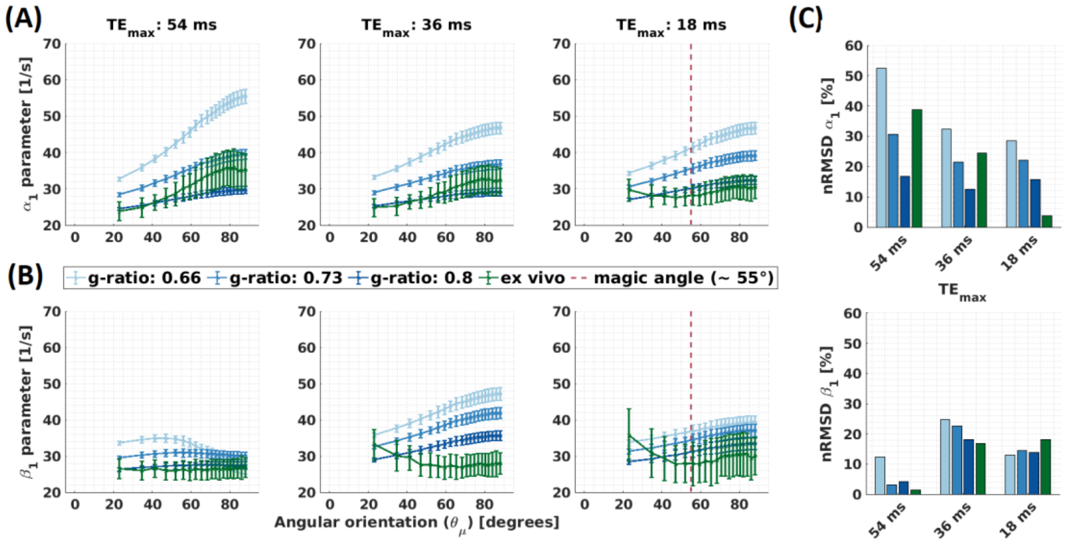

Figure S 7: Effect of the maximal echo time, i.e., meGRE subsets with different maximum echo times, on the $\theta_{\vec{\mu}}$ dependency of α_1 and β_1. (A and B) Angular orientation ($\theta_{\vec{\mu}}$) dependence of α_1 in M1 and β_1 in M2 for the three meGRE subsets with varying maximum TE (TE$_{max}$: 54 ms, 36 ms and 18 ms). Two datasets are compared: ex vivo (green curve) and in silico (blue curve) data at variable g-ratio; both only for the negligibly dispersed fibres ($\kappa \geq 2.5$). The magenta vertical lines in some of the subplots indicates the magic angle ($\theta_{\vec{\mu}}$ = 55°). (C) Quantification of the $\theta_{\vec{\mu}}$ dependency of α_1 and β_1 for three meGRE subsets with different maximal echo times (TE$_{max}$) by is the normalised root-mean-squared deviation (nRMSD, Equation 9 in %) of the α_1 parameter of M1 (proxy for R_2, top) and β_1 parameter of M2 (proxy for the isotropic part of R_2*, bottom). The distinct colours distinguish between in silico data at variable g-ratios (clear to dark blue bars) and ex vivo data (green bar), both for negligible dispersed fibres ($\kappa \geq 2.5$).*

7.2.4.2. Second sub-analysis: assessing if M2 is better explained by the data using meGRE subsets with different maximum echo times.

The average wAICc showed different trends across the different meGRE subsets with varying TE$_{max}$ for both datasets. For the *ex vivo* data, the average wAICc decreased when meGRE subsets with smaller TE$_{max}$ were used. Using the meGRE subsets with the largest and intermediate TE$_{max}$ (54 and 36 ms), the average wAICc indicated that M2 was better explained than M1 by the data with wAICc values in the ranges of wAICc > 0.73 (TE$_{max}$ = 54 ms) and 0.73 > wAICc > 0.5 (TE$_{max}$ = 36 ms), respectively. Interestingly, for the *in silico* data, the average wAICc depended on g-ratio when the meGRE subset with largest TE$_{max}$ was used (0.73 > wAICc > 0.5) but was below 0.5 and even non g-ratio dependent when the remaining meGRE subsets (the intermediate and lowest TE$_{max}$) were used (i.e., M2 was not better explained by the data than M1). Note that the large standard deviation of the reported wAICc per dataset indicates that the results are only valid on average whereas the wAICc for single voxels (*ex vivo* data) or replicas (*in silico* data) can be outside the reported ranges.

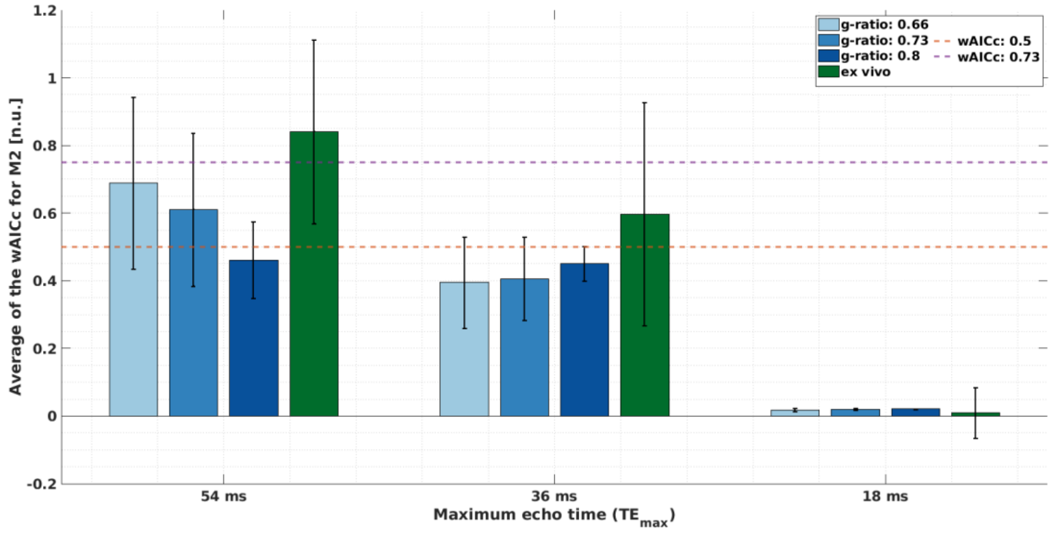

Figure S 8: Assessing if model M2 is better explained by the meGRE signal decay than M1, quantified by the averaged wAICc for M2 (Equation 12). This quantification was done per meGRE subsets with different maximum echo time (TE_{max}) for the in silico data at variable g-ratios (increased blue hue in bars, higher g-ratio) with R_2 values from Wharton and Bowtell, 2013; and ex vivo data (green bar) for negligibly dispersed fibres ($\kappa > 2.5$). The magenta and orange lines mark the following ranges: over the magenta line (wAICc = 0.73), M2 is better explained by the data; between the magenta and orange (wAICc = 0.5) lines, there is a preference for M2 but it is ambiguous whether M2 is better explained than M1 by the data; and bellow the orange line, M2 is not better explained by the data than M1.
